# When your host shuts down: larval diapause impacts host-microbiome interactions in *Nasonia vitripennis*

**DOI:** 10.1186/s40168-021-01037-6

**Published:** 2021-04-09

**Authors:** Jessica Dittmer, Robert M. Brucker

**Affiliations:** 1 The Rowland Institute at Harvard, Harvard University, 100 Edwin H. Land Boulevard, Cambridge, MA 02142 USA; 2 Present Address: Dipartimento di Scienze agrarie e ambientali (DISAA), Università degli Studi di Milano, Via Celoria 2, 20133 Milano, Italy

**Keywords:** Diapause, Gut microbiota, Metabolism, Nutrition, Symbiosis

## Abstract

**Background:**

The life cycles of many insect species include an obligatory or facultative diapause stage with arrested development and low metabolic activity as an overwintering strategy. Diapause is characterised by profound physiological changes in endocrine activity, cell proliferation and nutrient metabolism. However, little is known regarding host-microbiome interactions during diapause, despite the importance of bacterial symbionts for host nutrition and development. In this work, we investigated (i) the role of the microbiome for host nutrient allocation during diapause and (ii) the impact of larval diapause on microbiome dynamics in the parasitoid wasp *Nasonia vitripennis*, a model organism for host-microbiome interactions.

**Results:**

Our results demonstrate that the microbiome is essential for host nutrient allocation during diapause in *N. vitripennis*, as axenic diapausing larvae had consistently lower glucose and glycerol levels than conventional diapausing larvae, especially when exposed to cold temperature. In turn, microbiome composition was altered in diapausing larvae, potentially due to changes in the surrounding temperature, host nutrient levels and a downregulation of host immune genes. Importantly, prolonged larval diapause had a transstadial effect on the adult microbiome, with unknown consequences for host fitness. Notably, the most dominant microbiome member, *Providencia* sp., was drastically reduced in adults after more than 4 months of larval diapause, while potential bacterial pathogens increased in abundance.

**Conclusion:**

This work investigates host-microbiome interactions during a crucial developmental stage, which challenges both the insect host and its microbial associates. The impact of diapause on the microbiome is likely due to several factors, including altered host regulatory mechanisms and changes in the host environment.

**Video Abstract.**

**Supplementary Information:**

The online version contains supplementary material available at 10.1186/s40168-021-01037-6.

## Background

The life cycles of many insect species include an obligatory or facultative diapause stage as an adaptive strategy to increase survival during adverse environmental conditions [1]. In temperate climates, diapause is an overwintering strategy for insects to survive the cold season, analogous to hibernation in mammals and other types of dormancy, e.g., in plants. Diapause can occur at all life stages from the embryo to adulthood, but the exact stage is specific to each species. This diversity of diapause strategies in insects has undoubtedly contributed to their evolutionary success, allowing them to survive in fluctuating environments. Whereas some species have an obligatory diapause stage, recurring in each generation as an integral part of their life cycle, others can enter a facultative diapause depending on the prevailing environmental conditions. In the latter case, a sensitive developmental stage anticipates the arrival of unfavourable conditions via environmental cues and initiates preparation for diapause while conditions are still favourable. As such, the shorter daily photoperiods in late summer/autumn are token cues for upcoming seasonal change and trigger overwintering diapause [2–4].

Despite an increased interest in the regulatory and physiological aspects of insect diapause [1, 5–14], the dynamics of host-microbiome interactions during diapause remain underexplored (but see [15–18]). This contrasts with a growing body of research on hibernating mammals, demonstrating a seasonal remodelling of the gut microbiome between the hibernating and active phases in various species [19–22]. In brown bears, the gut microbiota also promotes the accumulation of fat stores in preparation for hibernation [22]. The scarcity of diapause-related host-microbiome studies is all the more surprising considering the importance of the microbiome for insect physiology. Notably, the microbiome promotes larval development and growth in numerous species by contributing to nutrient metabolism, gut homeostasis and endocrine signalling [23–29]. For instance, the gut microbiome regulates nutrient allocation in the fruit fly *Drosophila melanogaster* by modulating the expression of the insulin/insulin-like signalling pathway (IIS), a key regulator of insect growth and nutrient homeostasis [25]. Axenic fruit flies display increased lipid storage and hyperglycaemia, reminiscent of obesity and diabetes [27, 28]. This is of interest since sufficient nutrient reserves are crucial for survival during diapause and post-diapause fitness [2, 8, 30], and many insect species accumulate important nutrient reserves (e.g., lipids, storage proteins) during the preparation phase prior to diapause [7, 31]. Furthermore, *Burkholderia* gut symbionts increase juvenile hormone (JH) titres in the bean bug *Riptortus pedestris*, resulting in increased levels of storage proteins and egg production [32]. Hence, the gut microbiota interacts with the JH and the IIS pathways, which are likely key hormonal regulators of diapause induction and metabolic remodelling in diverse insects [6, 9, 12, 13].

Based on these observations, we hypothesised that the microbiome might play a role in regulating insect diapause, notably via its involvement in host nutrient allocation and metabolism. In turn, a host organism constitutes an ecosystem for its microbial associates [33], and therefore, changes in this ecosystem may impact the microbiome. For instance, certain microbiome members may not survive under diapause conditions [34] or enter into a state of reduced metabolic activity themselves, thereby enabling the overproliferation of other bacterial taxa. In combination with potentially altered host regulatory mechanisms, this may result in a deregulation of the microbiome during diapause, with unknown consequences for host survival during diapause and host fitness after diapause termination. From this perspective, insect diapause may present challenges for both the insect host and its microbial associates.

In the present work, we investigated the impact of diapause on host-microbiome interactions in the parasitoid wasp *Nasonia vitripennis*, an established model organism for host-microbiome interactions and diapause regulation [3, 35–40]. Indeed, *N. vitripennis* maintains a species-specific gut microbiome, which is essential for larval growth, successful pupation and survival to adulthood [23, 41–43]. In addition, short photoperiods induce a facultative overwintering diapause at the end of the final larval instar, immediately before pupation in non-diapausing individuals [3, 36, 44–46]. Larval diapause is maternally determined in this species, i.e., photoperiodic stimuli received by the mother will induce diapause in the progeny and, once “programmed”, no external stimuli received by the offspring can reverse this maternal predetermination [44, 45]. Interestingly, maternal diapause induction follows a “switch” pattern: When exposed to short photoperiods, females will produce non-diapausing offspring during the first days of adulthood, before rapidly switching to producing only diapause-destined offspring for the rest of their adult lifespan [44, 45]. The critical photoperiod and the number of daily cycles after which the switch occurs are genetically determined and represent local adaptations to seasonal cycles at different latitudes [3, 36, 44]. Herein, we leveraged this diapause “switch” to investigate nutrient allocation and microbiome composition in non-diapausing and diapausing siblings. Our results demonstrate that the microbiome is essential for nutrient allocation during diapause in *N. vitripennis*, especially in response to cold temperature. Furthermore, we reveal a transstadial effect of larval diapause on the adult microbiome, with as yet unknown consequences for host fitness.

## Results

### Larval diapause induction and termination in *N. vitripennis*

**Fig. 1:**
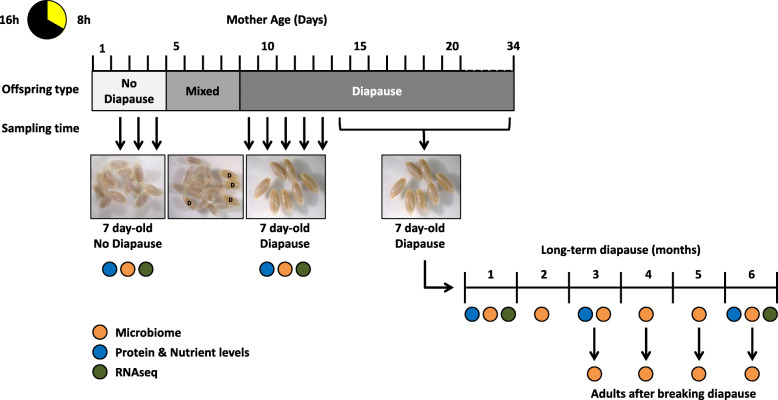
Experimental design. Diagram illustrating the “switch” to diapause induction in female *N. vitripennis* exposed to short photoperiods*.* This switch was leveraged to obtain final instar non-diapausing larvae and their age-matched diapause-destined siblings. The latter were then used in a diapause timecourse experiment. Coloured circles indicate which experiments were performed at each time-point. Diapause larvae in the picture of a mixed clutch are labelled with “D”

As previously shown [44, 45], *N. vitripennis* females exposed to diapause-inducing conditions (short photoperiod and 20°C) produced non-diapausing offspring in the first days of adult life before “switching” to the production of only diapause-destined offspring for the rest of their lifespan (Fig. 1). Depending on the female, the switch occurred 4–8 days after adult emergence, resulting in the production of mixed broods for only a few days (Fig. 1). This setup enabled us to obtain non-diapausing 4^th^ instar larvae and their age-matched diapausing siblings for subsequent experiments by sampling clutches of non-diapausing larvae from young females (2–5 days after adult emergence) and clutches of diapause-destined larvae from the same females a few days later (from 10 days after adult emergence onwards), when the wasp mothers had completed the “switch” and produced only 100% diapause-destined broods for the rest of their lives (Fig. 1). All larvae were reared at 25°C for 7 days until the last larval instar. After this period, both types of larvae have generally entirely ingested the fly host and stop feeding, as the non-diapausing larvae will soon pupate, and the diapause-destined larvae go into diapause. All non-diapausing and some of the diapause-destined larvae were sampled at this stage for subsequent analyses, but the majority of the diapause-destined larvae were maintained alive at 6°C and constant darkness for up to 6 months of diapause (Fig. 1). Larvae survival under these conditions was generally high: On average, 76% of the larvae in each clutch were still alive after 5 months and 74% after 6 months. Several diapausing larvae were transferred back to 25°C and long photoperiod every month to terminate diapause and resume development (Fig. 1). It was not possible to break diapause after the first 2 months, indicating that a minimum timespan in diapause or exposed to cold conditions is required before development can resume. Therefore, only post-diapause adults that had spent at least 3 months in larval diapause could be included in subsequent analyses.

### The microbiome is essential for host nutrient allocation during diapause

**Fig. 2:**
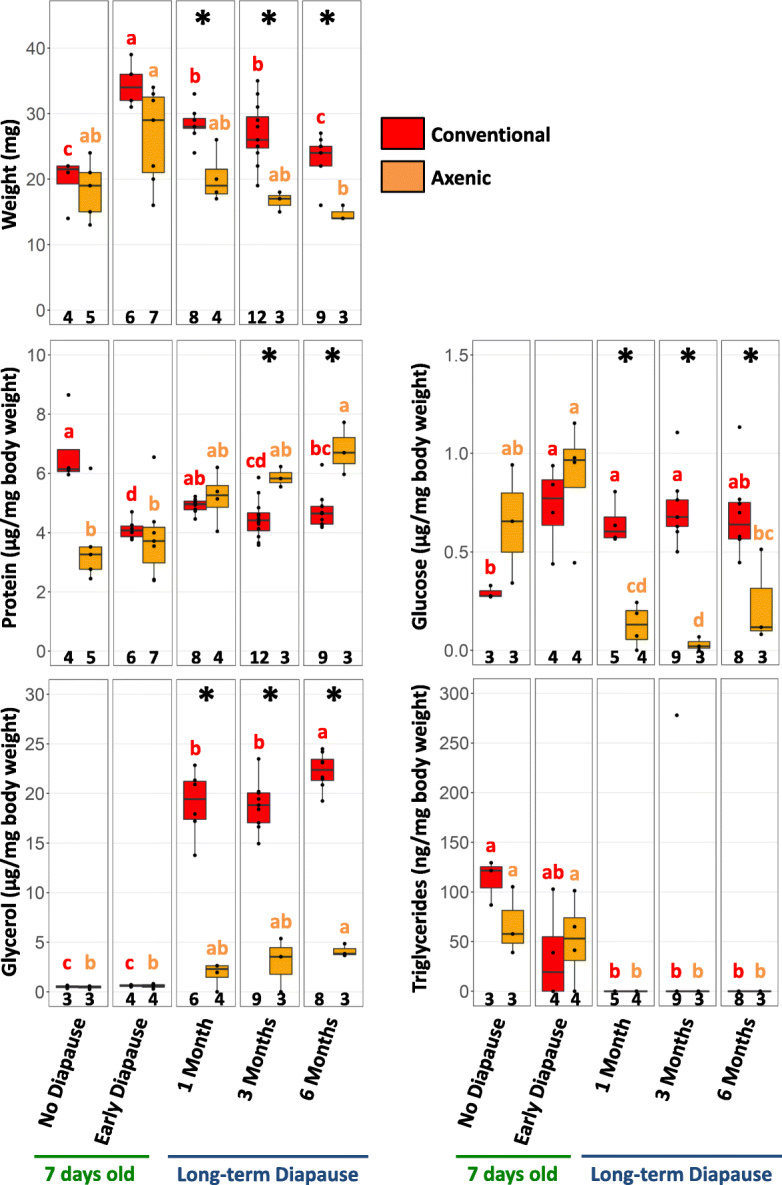
Impact of the microbiome on host nutritional reserves during larval diapause. Weight, total protein and nutrient levels were monitored in conventionally reared and axenic larvae at the end of larval development and over a 6-month period in diapause. Each datapoint represents a pool of 10 sibling larvae. Numbers at the bottom of each boxplot indicate the number of replicates per condition. Asterisks indicate significant differences between conventional and axenic larvae, based on pairwise Wilcoxon rank sum tests. Coloured letters indicate significant differences in weight, total protein and nutrient levels between experimental conditions for conventional (red) and axenic larvae (orange), based on Kruskal-Wallis rank sum tests followed by Dunn’s post hoc test with Benjamini-Hochberg correction for multiple comparisons. Different letters indicate significant differences between conditions

Considering that diapausing larvae need to survive for long periods of time without feeding, the establishment of sufficient nutrient reserves during the preparation for diapause and the economical use of these reserves throughout diapause is essential for host survival [8]. We investigated the role of the microbiome for host nutrient allocation in 4^th^ instar non-diapausing larvae, 4^th^ instar diapausing larvae (“early diapause”) as well as after 1, 3 and 6 months of diapause under cold conditions (6°C) (Figs. 1 and 2). For each condition, we measured total protein, glucose, glycerol and triglyceride levels in the same pools of 10 larvae (*N* = 3–12 replicate pools per time-point, Supplementary Table 1) and compared these nutritional levels between conventionally reared and axenic larvae. Axenic larvae were obtained from surface-sterilised eggs using the in vitro germ-free rearing method established for *Nasonia* [47, 48] and are therefore devoid of their microbiota. The surface sterilisation treatment constitutes an additional difference between the two groups, as it was only applied to the axenic larvae. Considering that axenic larvae weighed less than conventional larvae during diapause (Fig. 2), protein and nutrient levels were normalised by mg body weight to compare the two groups. Indeed, we observed only a transitory increase in weight in axenic early diapause larvae compared to axenic non-diapausing larvae. In contrast, conventionally reared early diapause larvae almost doubled in weight compared to age-matched non-diapausing larvae and maintained a higher weight for at least 3 months of diapause (Fig. 2).

For glucose, glycerol and triglycerides, nutrient levels were similar between conventional and axenic non-diapausing and early diapause larvae (Fig. 2), indicating no nutritional deficiencies due to the germ-free rearing medium, although these results should be interpreted with caution due to relatively low sample sizes for these conditions. In addition, we observed a tendency towards lower protein levels in axenic non-diapausing larvae compared to conventional larvae, but the difference was not statistically significant (Wilcoxon test, *W*=18, df=1, *p*=0.064). In conventional larvae, protein levels per milligram body weight were significantly lower in diapausing compared to non-diapausing larvae at all time-points except after 1 month in diapause (Kruskal-Wallis rank sum test with Dunn’s posthoc, all *p* < 0.008), demonstrating that protein levels did not increase linearly with body weight. In contrast, axenic larvae showed a gradual increase in protein levels per milligram body weight over time in diapause and had higher protein levels than conventionally reared larvae after 3 and 6 months of diapause (3 months: Wilcoxon test, *W*=2, df=1, *p*=0.018; 6 months: *W*=1, df=1, *p*=0.018) (Fig. 2). Considering that the body weight of axenic larvae remained mostly stable throughout the experiment, these results indicate a net increase in protein levels in axenic diapausing larvae.

Glucose levels increased by 2.5 fold in conventional early diapause larvae and remained high throughout the entire diapause period, whereas in axenic larvae, glucose levels declined from 1 month of diapause onwards, coinciding with cold exposure (Fig. 2). Thus, glucose levels of conventional larvae mirrored the pattern in weight gain very well. Similarly, conventional larvae experienced a 30-fold increase in glycerol levels under cold exposure from 1 to 6 months of diapause, while in axenic larvae, glycerol increased only slightly (Fig. 2). Consequently, axenic larvae had significantly lower glucose and glycerol levels compared to conventional larvae from 1–6 months in diapause (Wilcoxon test, all *p*≤0.024 (glucose) and all *p*≤0.012 (glycerol)). This is of interest as glycerol likely acts as a cryoprotectant in diapausing *Nasonia* [49] and the observed increase of glycerol in conventional larvae occurred after the transfer of the diapausing larvae from 25°C to 6°C for prolonged diapause. The coinciding increase in glucose levels may be explained by the breakdown of glycogen into glucose, which is then used for glycerol synthesis in response to low temperatures [8]. In addition, increased glycolysis/gluconeogenesis has been associated with a switch to anaerobic metabolism during diapause in several dipterans [5, 12, 50, 51]. In contrast, triglycerides, the major storage form of lipids in insects, were generally low in both conventional and axenic larvae and dropped even lower after diapause initiation (Fig. 2). These observations indicate that the microbiome plays an essential role in nutrient allocation, mobilisation and metabolism during diapause in *N. vitripennis*, notably when exposed to cold temperature.

### Microbiome dynamics during larval diapause

**Fig. 3:**
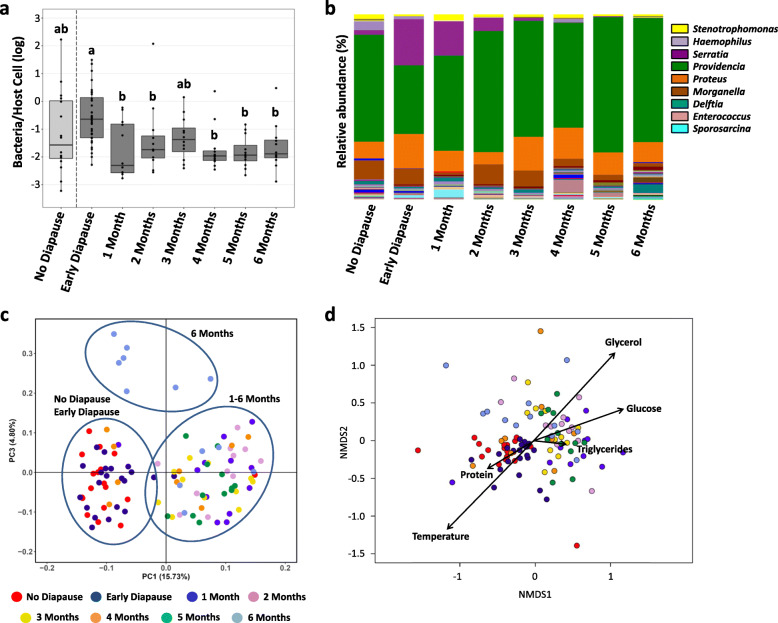
Microbiome dynamics during larval diapause. **a** Bacterial titres in non-diapausing larvae, their age-matched early diapause siblings and in diapausing larvae over a 6-month timecourse. Each datapoint represents a pool of 5–12 larvae (Suppl. Table 3). Different letters indicate significant differences between experimental conditions based on Kruskal-Wallis rank sum test and Dunn’s post hoc test for multiple comparisons. **b** Microbiome composition per experimental condition. The most abundant bacterial genera are represented in the legend. Microbiome composition in each replicate sample is provided in Supplementary Figure S1. **c** Principal Coordinates Analysis based on unweighted unifrac distances showing differences in microbiome composition depending on diapause. The datapoints correspond to the same sibling pools as in (**a**). **d** Nonmetric Multidimensional Scaling based on Bray-Curtis distances between bacterial communities demonstrating the correlation between changes in microbiome composition and environmental factors, i.e., temperature and host nutrient levels. Datapoints are colour-coded by experimental condition as in (**c**)

Diapause-related changes in bacterial titres and microbiome composition were investigated using quantitative PCR and 16S rRNA gene amplicon sequencing, respectively, on the same biological samples (Supplementary Tables 2, 3 and 4). These experiments were performed on 4^th^ instar non-diapausing larvae, 4^th^ instar early diapause larvae as well as over a 6-month diapause period under cold exposure (*N*=9–21 replicate pools of 5–12 sibling larvae per condition) (Figs. 1and 3). Overall, bacterial titres were stable and not significantly different between diapausing and non-diapausing larvae (Fig. 3a). However, larvae after 1–6 months of diapause had generally lower bacterial titres compared to early diapause larvae (Kruskal-Wallis rank sum test followed by Dunn’s post hoc, all *p*<0.02), except after 3 months of diapause (Kruskal-Wallis rank sum test followed by Dunn’s posthoc, *p*=0.12) (Fig. 3a).

Amplicon sequencing produced 5589–37394 high-quality reads per sample, which clustered into 792 OTUs (19-152 OTUs per sample, mean=65 OTUs) based on 97% similarity (Supplementary Tables 3, 4). In line with previous studies [40, 42, 43], the genus *Providencia* was the most dominant member of the larval microbiome (56.78% of all reads), followed by *Proteus* (12.95%), *Serratia* (8.78%) and *Morganella* (6.69%) (Fig. 3b, Supplementary Fig. S1). Together, these four gammaproteobacterial taxa accounted for 85% of the larval microbiome across all experimental conditions. The remaining OTUs represented 7 bacterial phyla: Acidobacteria (2 genera), Actinobacteria (39 genera), Bacteroidetes (14 genera), Firmicutes (54 genera), Fusobacteria (3 genera), Gemmatimonadetes (1 genus) and Proteobacteria (79 genera). Bacterial species richness and diversity as measured using the species richness estimator Chao1 and the Shannon Index of diversity were mostly unaffected by larval diapause, except for a decreased species richness in larvae after 2 months of diapause compared to early diapause larvae (*t* test, *t*=−3.61, df=1, *p*=0.028) (Supplementary Fig. S2).

Whereas bacterial titres and species richness remained mostly stable, bacterial community composition changed throughout the experimental time course, as indicated by Adonis and Anosim based on unweighted unifrac distances between experimental conditions (Adonis: *F*=3.63, *R*^2^=0.21, *p*=0.0001; Anosim: *R*=0.46, *p*=0.0001). Specifically, the microbiomes of non-diapausing larvae and their age-matched early diapause siblings clustered together in a PCoA analysis, while most of the prolonged diapause samples that were exposed to cold temperature (1–6 months of diapause) formed a second cluster (Fig. 3c). Interestingly, more than half of the 6-month-diapause samples formed a separate, more distant cluster (Fig. 3c), indicating that diapause-induced changes in larval microbiome composition may only appear after several months in diapause. To identify factors correlated with the differences in microbiome structure, we analysed the impact of host physiology (i.e., protein and nutrient levels) and the surrounding environment (i.e., temperature) using the EnvFit function of the R package “vegan”. Indeed, both host nutrient levels and temperature were significantly correlated with changes in microbiome composition (temperature: *r*^2^=0.4072, *p*=0.001; glycerol: *r*^2^=0.4055, *p*=0.001; glucose: *r*^2^=0.2524, *p*=0.001; protein: *r*^2^=0.0745, *p*=0.018; triglycerides: *r*^2^=0.0817, *p*=0.019) (Fig. 3d). Specifically, temperature was positively correlated with the cluster corresponding to non-diapausing and early diapause larvae and negatively correlated with the cluster corresponding to prolonged diapause. Glycerol was inversely correlated with temperature, consistent with glycerol acting as a cryoprotectant in response to the lower rearing temperature. On the other hand, protein and glucose levels changed already in early diapause larvae (i.e., prior to cold exposure, Fig. 2); therefore, their effect on microbiome composition was likely independent from the temperature effect. These results indicate that microbiome composition changes in diapausing larvae were strongly correlated with host nutrient levels and the surrounding temperature.

**Fig. 4:**
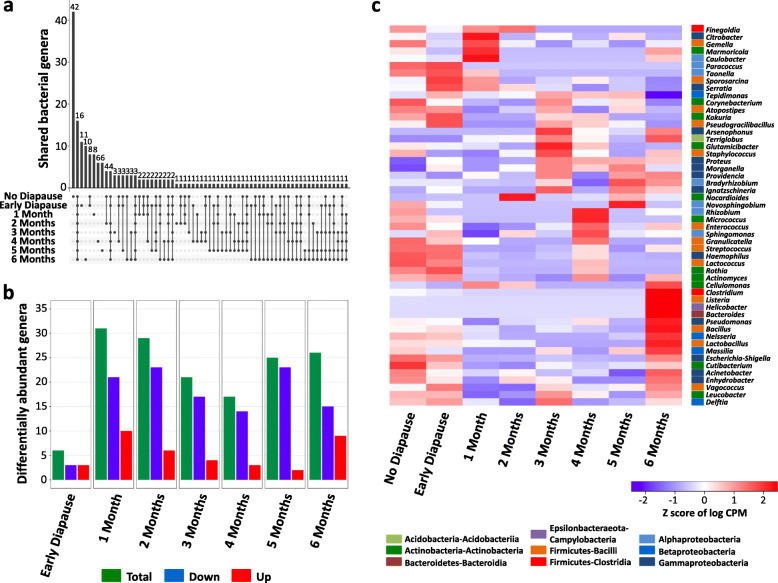
Differentially abundant genera during larval diapause. **a** Intersection plot showing the shared and specific bacterial genera depending on diapause conditions. The lower part of the plot shows in which experimental conditions (indicated by dots connected by a line) the different genera were observed. **b** Numbers of differentially abundant bacterial genera in diapausing compared to non-diapausing larvae. The majority of differentially abundant taxa decrease in abundance with prolonged larval diapause. **c** Heatmap showing the abundance of 52 bacterial genera throughout the experimental timecourse. Each genus was differentially abundant in at least one diapause condition compared to non-diapausing larvae. Coloured boxes preceding genus names indicate bacterial phylum and class. Differences in abundance are indicated by the *z* score of log CPM (counts per million) values. Rows are ordered based on abundance patterns using hierarchical clustering. Biological replicates per experimental condition were grouped together for readability. The full heatmap is provided in Supplementary Figure S3

Subsequently, we identified the bacterial genera which changed in abundance between experimental conditions. Only 16 bacterial genera (out of 193, i.e., 8.3%) were consistently present across all experimental conditions (Fig. 4a, Supplementary Table 4). Apart from the four aforementioned dominant taxa *Providencia*, *Proteus*, *Serratia* and *Morganella*, these included 12 low-abundance genera belonging to the Actinobacteria (*Corynebacterium*, *Nocardioides*, *Cutibacterium*), Firmicutes-Bacilli (*Sporosarcina*, *Staphylococcus*, *Streptococcus*), Alphaproteobacteria (*Methylobacterium*), Betaproteobacteria (*Achromobacter*, *Delftia*, *Ralstonia*) and Gammaproteobacteria (*Haemophilus*, *Stenotrophomonas*). Despite the low abundance of all remaining genera (177 genera accounting for only 4.84% of all reads), it is noteworthy that 42 of these genera were exclusively present in non-diapausing larvae and absent from diapausing larvae, whereas only 4–11 genera were exclusively present at different diapause time-points (Fig. 4a). This indicates that some genera may be lost already during diapause preparation, as they were also absent from 4^th^ instar early diapause larvae.

In addition, 52 genera were differentially abundant in at least one diapause time-point compared to non-diapausing larvae (Fig. 4b,c; Supplementary Fig. S3; Supplementary Table 4). While only six genera were differentially abundant in early diapause larvae, 17–31 genera were differentially abundant after 1–6 months in diapause (Fig. 4b). The majority of these were less abundant in diapausing compared to non-diapausing larvae (Fig. 4b), indicating that some bacterial genera may decline with prolonged diapause, potentially due to lack of nutrients or the low-temperature environment. Nonetheless, several genera increased in abundance at particular time-points (Fig. 4c). Genera which increased in abundance over a longer period of time (i.e., at least 3 months of the 6-month diapause period) included the male-killing endosymbiont *Arsenophonus nasoniae* (increased after 3, 4 and 6 months of diapause; log_2_ fold change: 3–8), the gammaproteobacterium *Ignatzschineria* (increased after 3, 5 and 6 months; log_2_ fold change: 6) and the acidobacterium *Terriglobus*, which was more abundant at all time-points from 1–6 months in diapause (log_2_ fold change: 2.3–7.5) compared to non-diapausing larvae (Fig. 4c). This genus consists of widespread aerobic soil bacteria, able to survive under low nutrient, low temperature, low oxygen and slightly acidic conditions [52, 53], which may explain its proliferation in the gut of diapausing larvae. In addition, the genera *Bacteroides*, *Helicobacter*, *Listeria*, *Vagococcus*, *Clostridium* and *Neisseria* had significantly higher abundances (log_2_ fold changes from 3.7 to 11), specifically after 6 months of diapause (Fig. 4c). Interestingly, these genera increased in abundance in 5–8 out of 12 biological replicates (Supplementary Fig. S3), and the samples with increased abundances of these taxa corresponded to those forming the distinct 6-month-diapause cluster (Fig. 3c).

### Changes in host regulatory mechanisms during larval diapause

**Fig. 5:**
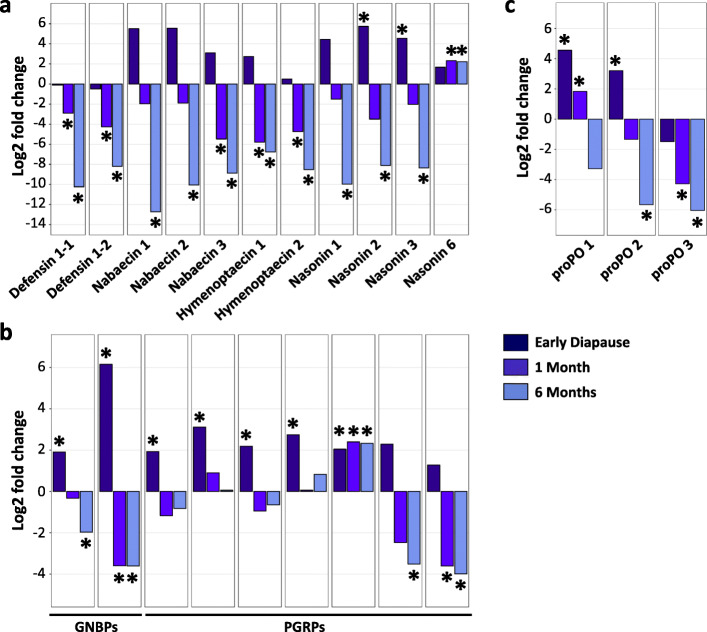
Downregulation of immunity-related genes during larval diapause. Log_2_ fold changes of genes coding for **a** antimicrobial peptides, **b** proPhenoloxidases and **c** pattern recognition proteins (Gram-negative binding proteins (GNBPs) and Peptidoglycan recognition proteins (PGRPs)) in diapausing larvae compared to non-diapausing larvae. Asterisks indicate significant differences in gene expression after FDR correction. Most genes were significantly downregulated after prolonged larval diapause

To investigate the role of host regulatory mechanisms in driving the observed changes in the larval microbiome during diapause, we performed RNAseq on 4^th^ instar non-diapausing larvae, 4^th^ instar early diapause larvae and diapausing larvae after 1 and 6 months of diapause. Three biological replicates (i.e., pools of 10 larvae) were used per condition and corresponded to larvae samples also included in the qPCR and amplicon sequencing analyses outlined above. The number of differentially expressed genes in diapausing larvae compared to non-diapausing larvae increased with diapause duration, ranging from 999 differentially expressed genes in early diapause larvae and 1177 after 1 month of diapause to 2438 after 6 months of diapause. Considering that the host’s immune response is a key regulator of microbiome composition [54, 55], e.g., by preventing the overproliferation of certain taxa, we searched the differentially expressed genes and identified 23 immunity-related genes which were differentially expressed in at least one diapause time-point: 11 genes coding for antimicrobial peptides, three proPhenoloxidases (proPO) potentially involved in the melanisation of foreign particles and nine pattern-recognition proteins (two gram-negative binding proteins (GNBPs) and seven peptidoglycan recognition proteins (PGRPs)) (Table 1; Fig. 5). Importantly, all but one of the antimicrobial peptides and two out of three proPOs were significantly downregulated with prolonged diapause. Only the antimicrobial peptide Nasonin 6 remained upregulated after 1 and 6 months of diapause. Both GNBPs and five PGRPs were initially upregulated in early diapause larvae, but their expression levels gradually declined during diapause, resulting in both GNBPs and two PGRPs being downregulated after 6 months of diapause (Table 1; Fig. 5). These results indicate that the host’s immune system is strongly repressed during diapause, which may have contributed to the deregulation of the microbiome.

**Table 1:** Differentially expressed immunity genes in diapausing larvae compared to non-diapausing larvae. Downregulated genes are highlighted in bold

Gene	Locus^**a**^	Early diapause		1 month		6 months	
		Log_**2**_ fold change	Adj.^**b**^ ***p*** value	Log_**2**_ fold change	Adj.^**b**^ ***p*** value	Log_**2**_ fold change	Adj.^**b**^ ***p*** value
**Antimicrobial peptides**							
Defensin 1-1	LOC100115742			**−2.91**	0.016	**−10.24**	1.54E**−**15
Defensin 1-2	LOC100310843			**−4.27**	0.00016	**−8.20**	4.67E**−**12
Nabaecin 1	LOC100117200					**−12.73**	0.0012
Nabaecin 2	LOC100422736					**−10.06**	0.0018
Nabaecin 3	LOC100678093			**−5.48**	0.0092	**−8.88**	0.0001
Nahymenoptaecin 1	LOC100122694			**−5.79**	0.00062	**−6.77**	3.52E**−**05
Nahymenoptaecin 2	LOC100116503			**−4.73**	6.86E**−**05	**−8.52**	6.72E**−**12
Nasonin 1	LOC100120544					**−9.97**	0.00022
Nasonin 2	LOC100462678	5.74	0.0014			**−8.11**	2.53E**−**05
Nasonin 3	LOC100123729	4.55	0.028			**−8.35**	2.34E**−**05
Nasonin 6	LOC100114068			2.32	0.0091	2.22	0.0082
**Phenoloxidases**							
ProPO	LOC100122972	4.56	1.87E**−**06	1.83	0.019		
ProPO	LOC100116823	3.21	0.00087			**−5.66**	1.91E**−**08
ProPO	LOC100121103			**−4.28**	1.24E**−**06	**−6.04**	4.33E**−**09
**GNBPs**							
GNBP 1-2	LOC100123967	1.91	0.0035			**−1.97**	0.0012
GNBP 2	LOC100313520	6.16	7.24E**−**15	**−3.60**	0.0005	**−3.61**	0.00033
**PGRPs**							
PGRP	LOC100117008	1.93	0.026				
PGRP	LOC100117452	3.12	6.57E**−**06				
PGRP	LOC100119736	2.19	0.0029				
PGRP	LOC100119764	2.75	0.023				
PGRP	LOC100119797	2.05	0.048	2.40	0.0046	2.33	0.0039
PGRP	LOC100121589					**−3.51**	0.021
PGRP	LOC100121609			**−3.61**	0.0032	**−3.99**	0.0005

^**a**^Locus tags are based on the *N. vitripennis* genome assembly 2.1 (Accession No. GCA_000002325.2)

^**b**^*p* values were FDR-corrected using the Benjamini-Hochberg algorithm

### A transstadial effect of larval diapause on the adult microbiome

**Fig. 6:**
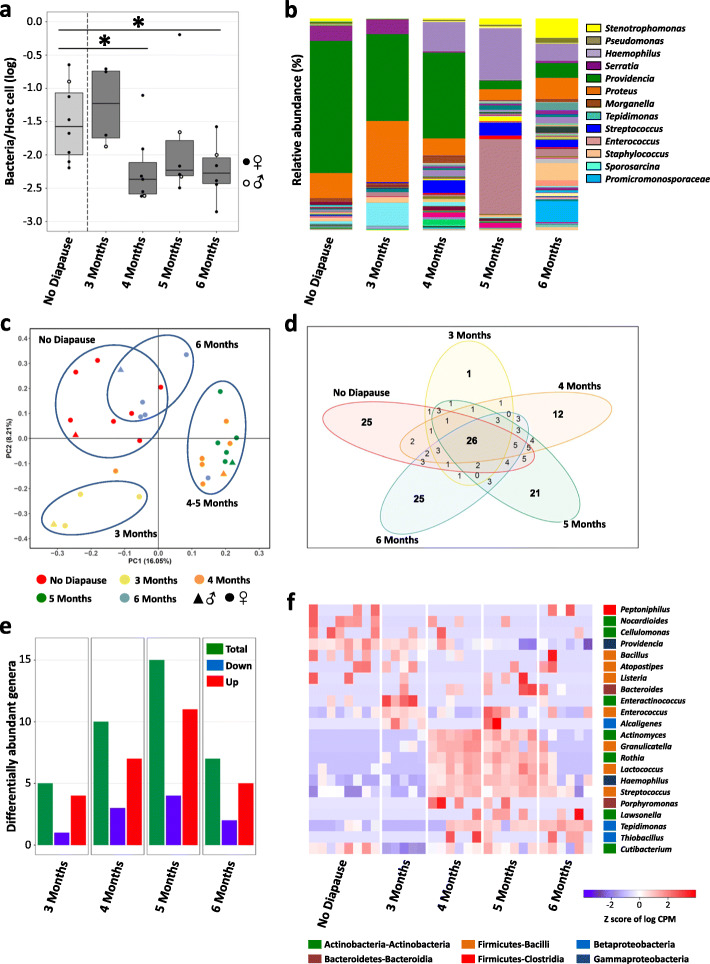
Transstadial effect of larval diapause on adult microbiome. **a** Bacterial titres in newly emerged adults after direct development (no diapause) and in their siblings after different periods of larval diapause. Each datapoint represents a pool of 2–6 siblings (Suppl. Table 3). Closed circles represent pools of females, open circles represent pools of males. Asterisks indicate significant differences between adults after direct development and post-diapause, based on pairwise Wilcoxon rank sum tests. **b** Microbiome composition per experimental condition. The most abundant bacterial genera are represented in the legend. Microbiome composition in each replicate sample is provided in Supplementary Figure S4. **c** Principal Coordinates Analysis based on unweighted unifrac distances showing differences in microbiome composition depending on diapause duration. The datapoints correspond to the same sibling pools as in (**a**). Circles represent pools of females, triangles represent pools of males. **d** Venn diagram showing the shared and specific bacterial genera depending on experimental conditions. **e** Numbers of differentially abundant bacterial genera in post-diapause adults compared to directly developed adults. **f** Heatmap showing the abundance of 22 bacterial genera throughout the experimental timecourse. Each genus was differentially abundant in at least one post-diapause condition compared to directly developed adults. Coloured boxes preceding genus names indicate bacterial phylum and class. Differences in abundance are indicated by the *z* score of log CPM (counts per million) values. Rows are ordered based on abundance patterns using hierarchical clustering

Next, we investigated whether larval diapause had an effect on the adult microbiome. To this end, we compared the microbiome of newly emerged adults after diapause termination (post-diapause adults) to the adult microbiome after direct development without a diapause stage (non-diapause adults). Bacterial titre quantification and 16S rRNA gene amplicon sequencing were performed on the same pools of 2–6 adults (*N*=4–8 replicate pools per condition, Supplementary Tables 2, 3 and 4). Bacterial titres were significantly lower in post-diapause adults compared to non-diapause adults after 4 months and 6 months of diapause (4 months: Wilcoxon test, *W*=9, df=1, *p*=0.032; 6 months: *W*=7, df=1, *p*=0.029) (Fig. 6a). Amplicon sequencing produced 3286–22852 high-quality reads per sample, which were clustered into 693 OTUs (41-123 OTUs per sample, mean=80 OTUs) (Supplementary Tables 3, 4). Bacterial species richness was significantly higher in adults after 3 months of diapause compared to non-diapause adults (*t* test, *t*=−4.48, df=1, *p*=0.02) but was not significantly different in adults after longer periods of diapause. In contrast, bacterial diversity increased with diapause duration and was significantly higher after 6 months of diapause compared to 3 months of diapause (*t* test, *t*=10.01, df=1, *p*=0.01) (Supplementary Fig. S2). This suggests that the bacterial taxa were more evenly represented in the adult microbiome after longer periods of larval diapause.

In addition, diapause duration had a stronger effect on microbiome composition in adults than in larvae, since the microbiomes of post-diapause adults formed distinct clusters from the microbiomes of non-diapause adults. Furthermore, the post-diapause clusters reflected diapause duration (Fig. 6c), indicating gradual changes in microbiome composition depending on diapause duration. This was due to numerous genera that occurred specifically in non-diapause vs. post-diapause adults or changed in abundance depending on diapause duration. Hence, 25 out of 168 identified genera (14.9%) were only present in non-diapause adults (Fig. 6d). While only one genus was specifically associated with adults after 3 months of diapause, the number of specific genera increased with diapause duration (Fig. 6d). This indicates that, as in larvae, some bacterial taxa may be lost from (or no longer transmitted to) the adult microbiome, even after short periods of larval diapause. However, in contrast to larvae, prolonged diapause also resulted in the detection of new taxa in the adult microbiome. It remains to be determined whether these bacteria were newly acquired from the environment after diapause termination or whether they had increased in abundance from previously undetectably low levels, due to the availability of new niches after the disappearance of diapause-sensitive taxa.

In addition, 22 genera were differentially abundant in at least one post-diapause time-point compared to non-diapause adults (Fig. 6e,f; Supplementary Table 4). In contrast to larvae, the majority of these genera increased in abundance in post-diapause adults (Fig. 6e). Notably, the genera *Actinomyces*, *Rothia* (both Actinobacteria), *Granulicatella*, *Lactococcus*, *Streptococcus* (Bacilli) and *Haemophilus* (Gammaproteobacteria) increased in abundance in all biological replicates after 4–5 months of diapause (Fig. 6f) with log_2_ fold changes of 3.65–11.43. In terms of relative abundance, *Haemophilus* accounted for less than 0.5% of the microbiome in non-diapause adults but represented 14, 24 and 8% of the microbiome after four to 6 months of diapause, respectively (Fig. 6b). Similarly, *Streptococcus* increased from less than 0.5% in non-diapause adults to 6% of the microbiome after 4–5 months of diapause (Fig. 6b). There was also an important temporary increase of *Enterococcus* after 5 months of diapause, but this bacterium was abundant in only 50% of the samples (Fig. 6b,f, Supplementary Fig. S4). Interestingly, most of these taxa had significantly decreased in abundance in diapausing larvae compared to non-diapausing larvae (Fig. 4c), indicating that the changes in abundance in the post-diapause adult microbiome did not simply reflect previous changes in the larval microbiome.

In contrast, *Providencia* was by far the most dominant member of the adult microbiome after direct development (62.43% of all reads) (Fig. 6b, Supplementary Fig. S4). However, its relative abundance decreased strongly in post-diapause adults, accounting for 40–41% of the microbiome after 3–4 months of diapause, and for only 4–7% of the microbiome after 5–6 months of diapause (Fig. 6b; Supplementary Fig. S4). This corresponded to a significant decrease in abundance after 5–6 months of diapause (log_2_ fold change of −4.63 and −5.18, respectively) (Fig. 6f). Hence, the most dominant member of the *Nasonia* microbiome was most strongly affected by prolonged larval diapause.

## Discussion

Diapause is not simply a developmental and metabolic arrest but a physiologically dynamic alternative stage to direct development [1, 8, 10]. It is accompanied by profound changes in endocrinal activity [11–13], cell proliferation [46], stress resistance and nutrient metabolism [7, 12, 14, 31, 49, 51, 56]. In the present work, we demonstrate that the microbiome is an additional factor contributing to the diapause-associated metabolic changes. Indeed, the microbiome played an important role in nutrient allocation and mobilisation during larval diapause in the parasitoid wasp *N. vitripennis*, since axenic larvae accumulated less weight and had consistently lower glucose and glycerol levels throughout diapause than conventional larvae, especially when exposed to cold temperature. Considering that glycerol acts as a cryoprotectant [57], it is important to note that axenic larvae survived cold exposure during the 6-month experimental period despite their low glycerol levels, likely because *Nasonia* larvae can withstand much lower temperatures than the one applied here [57]. The increase in glucose levels could be explained by the transition to anaerobic metabolism with increased glycolysis/gluconeogenesis during diapause, as observed in several dipterans [5, 12, 50, 51]. If this were the case, then the microbiome would be required to maintain glycolysis/gluconeogenesis during cold exposure in *N. vitripennis*. In contrast, total protein levels gradually increased in axenic diapausing larvae under the same conditions. While the proteins were not further identified in this study, it is tempting to speculate whether these proteins might be storage proteins such as hexamerins. These results demonstrate that the microbiome may be involved in the metabolic remodelling and cold resistance associated with diapause, which may have important consequences for host survival of diapause and post-diapause fitness. Deciphering the underlying mechanisms and the role of specific bacterial taxa in mediating this interaction will be important avenues for future research into diapause regulation.

In turn, larval diapause altered larval microbiome composition over time. While the dominant members of the microbiome remained largely unaffected, diapause seemed to have an impact on many less abundant taxa. This is similar to observations in stinkbugs, where only a non-transient core microbiome was detected in diapausing individuals [17]. In *N. vitripennis*, about 20% of all bacterial genera were exclusively observed in non-diapausing larvae, indicating that some bacterial taxa may be lost already during preparation for diapause. *Nasonia* acquires its microbiome by feeding on its fly host, which is inoculated with bacteria from the mother. This makes environmental filtering of certain bacteria by diapause-destined larvae unlikely, as they can be assumed to ingest the same source bacteria as their non-diapausing siblings. Alternatively, certain bacteria may not persist in the altered host environment, due to changes in physiological conditions or the type of available nutrients. For instance, bacterial taxa known to depend on dietary polysaccharides were found to decline in hibernating mammals, while bacteria able to degrade host-derived nutrients increased in abundance during hibernation [19–22]. Subsequently, a large fraction of the remaining bacterial genera decreased in abundance with prolonged diapause, while only a few genera increased in abundance. This decrease may again be due to reduced microbial fitness of certain bacteria, as previously observed for the intracellular symbiont *Wolbachia* [34]. An alternative explanation might be that the more sensitive taxa survive, but enter into a state of reduced metabolic activity and reduced multiplication in response to low environmental temperatures or changes in available nutrients. The latter explanation was further supported by our observation that the changes in microbiome composition were strongly correlated with changes in host nutrient levels and the surrounding temperature. The latter is reminiscent of a previously observed shift in microbiome composition under low-temperature conditions in diapausing crickets [18]. Specifically, the “diapause microbiome” of *N. vitripennis* was associated with lower temperature and lower protein levels, but higher glucose and glycerol levels. It is important to note that the increase in glycerol was itself driven by the low temperature and not by the diapause state itself. Consequently, environmental temperature and changes in host nutrient metabolism impacted microbiome composition synergistically.

Nonetheless, a few bacterial genera increased in abundance, especially after 6 months of diapause. These bacteria included common inhabitants of animal guts (e.g., *Bacteroides, Clostridium*), the genus *Terriglobus* known to thrive in low temperature and nutrient-poor environments [52, 53] and the male-killing symbiont *Arsenophonus nasoniae* [58–61]. The increase of these taxa may have been facilitated by their metabolic versatility, the availability of new ecological niches due to the reduction of diapause-sensitive taxa and/or reduced host regulatory mechanisms. Indeed, numerous immunity-related genes coding for antimicrobial peptides, proPhenoloxidases and pattern recognition proteins were severely repressed, especially after 6 months of diapause. This relaxation of host regulatory mechanisms may have contributed to the overproliferation of certain taxa or to the tolerance of the altered microbiome by the host. Although the precise interplay between these genes and the microbiome remains to be investigated, it is likely that the downregulation of numerous immunity-related genes is relevant for the host-microbiome cross-talk, since previous work demonstrated an upregulation of immunity-related genes in interspecies hybrids with altered microbiomes [42]. Importantly, the antimicrobial peptide Nasonin 6 and one PGRP were consistently upregulated in diapausing larvae, reinforcing the perspective that diapause is not a complete physiological shutdown, but rather a physiologically remodelled developmental state [1, 8]. A previous study on diapause-related changes in the microbiome reported an increased abundance of numerous bacteria during the preparation for adult diapause in the cabbage beetle *Colaphellus bowringi* and this increase correlated with increased lipid stores [15]. In contrast, changes in microbiome composition were not necessary to induce the metabolic changes leading to diapause in *N. vitripennis*, since the nutritional state was already different in early diapause larvae whose microbiome was still similar to the microbiome of non-diapausing (i.e., not fat-accumulating) larvae. Rather, the presence of the microbiome was important for host nutrient allocation during diapause, as indicated by the comparison of nutrient stores in conventional vs. germ-free larvae. These differences in nutrient allocation and the impact of diapause on the microbiome between the two organisms clearly highlight the need for more comprehensive studies integrating host metabolism and the microbiome in insects with different diapause stages.

Larval diapause had a transstadial effect on the adult microbiome, especially after more than 3 months of diapause. In contrast to larvae, numerous taxa increased in abundance in the adult microbiome after prolonged diapause, with the notable exception of *Providencia*, which is usually the most dominant bacterium in the microbiome of adult *N. vitripennis* [42, 43]. Consequently, the changes in the adult microbiome did not simply reflect previous changes in the larval microbiome. Under non-diapause-inducing conditions, *N. vitripennis* experiences a microbial succession throughout its development [43]. Notably, bacterial diversity increases during the non-feeding pupal and adult stages, but the mechanisms driving these changes in the microbiome during metamorphosis have not been elucidated. We hypothesise that the observed changes in the post-diapause adult microbiome reflect a deregulation of the microbial successions during metamorphosis compared to non-diapausing individuals. This may be a consequence of (i) altered host regulatory mechanisms such as immunity, (ii) an altered host environment (e.g., due to changes in nutrients carried over from the larval stage) favouring some bacterial taxa but not others and/or (iii) altered microbial fitness, depending on the duration of larval diapause. The observed impact of larval diapause on the adult microbiome may have practical implications, as *Nasonia* is reared for the biological control of filth flies and diapause is often exploited as a shipping or storage strategy by commercial vendors and research labs. Considering that very little is known about the functional roles of specific taxa within the *Nasonia* microbiome (but see [40]), the potential impact of the diapause-induced changes in the microbiome on adult fitness remains as yet unknown. While all genera which increased in abundance are common members of the commensal gut microbiota in many species, some (e.g., *Clostridium* or *Enterococcus*) may become opportunistic pathogens if not kept in check. More importantly, the functional role of *Providencia*, normally the most abundant taxon but drastically reduced after prolonged diapause, is not yet understood. In other organisms, *Providencia* species have been shown to provide B vitamins [62] or to protect against bacterial pathogens [63]. Other *Providencia* species may be pathogenic; however, the consistent predominance of this taxon in healthy *N. vitripennis* makes a pathogenic relationship in this species unlikely. Finally, it remains to be tested whether the diapause-induced shift in the adult microbiome is permanent or whether it can be reversed over time, e.g., when the female gets in contact with the microbiota of a fly host during oviposition.

This study used a *Wolbachia*-cured laboratory line to investigate diapause-related changes even in minor taxa within the microbiome, which would otherwise have been masked by the dominant *Wolbachia*. However, wild *N. vitripennis* harbour two *Wolbachia* strains whose impacts on microbiome composition have not been investigated to date. Considering that both *Wolbachia* strains appear to be sensitive to prolonged diapause (or the associated cold temperature) under laboratory conditions [34], a reduction of *Wolbachia* titres with prolonged diapause might open up new niches and resources for the rest of the microbiome. The dynamics of *Wolbachia*-microbiome interactions during diapause thus represent an interesting avenue for future research.

## Conclusions

This work investigates host-microbiome interactions during insect diapause, a crucial developmental stage which challenges both the insect host and its microbial associates. To our knowledge, this is the first demonstration of a functional role of the microbiome for nutrient allocation and mobilisation during diapause, indicating that the microbiome should be taken into account as an additional factor determining diapause physiology. In turn, larval diapause altered the microbiome of diapausing larvae and post-diapause adults, with unknown consequences for host fitness.

## Methods

### Experimental design

The *Nasonia vitripennis* wasps used in this work came from the *Wolbachia*-cured laboratory line AsymCx. Wasps were reared on *Sarcophaga bullata* pupae at 25°C and a long photoperiod (15 h of light). For diapause induction, our experimental design was inspired by the early work by Saunders [44, 45]. Twelve to 13 days after hosting, fly host puparia were opened and *Nasonia* black pupae were sorted into mating pairs consisting of one male and one female. Each mating pair was placed into an insect vial and maintained at 25°C and long photoperiod until emergence as adults. Upon emergence, the insect vials were transferred to diapause-inducing conditions (20°C and a short photoperiod of 8 h of light) and two *S. bullata* pupae were given to each mating pair to allow for oviposition. The two fly hosts were replaced every 24h throughout the entire lifespan of the female wasps. The parasitized hosts were maintained at 25°C for optimal larval development and were opened after 7 days to collect *N. vitripennis* 4th instar larvae, thereby obtaining daily larvae clutches from each individual female. Since *N. vitripennis* females in these conditions produce non-diapausing offspring during the first days of adult life and then switch to producing diapause-destined offspring for the rest of their life [44, 45], this setup enabled us to obtain non-diapausing larvae and their age-matched diapause-destined siblings. We found that diapausing and non-diapausing larvae cannot be reliably distinguished by eye; therefore, several larvae from each clutch were kept alive to check for pupation a few days later. To achieve this, the larvae were stuck on double-sided tape in a petri dish and maintained at 25°C. This allowed us to determine non-diapausing, diapausing and mixed broods a posteriori. The remaining individuals from each larval clutch were either (i) immediately frozen at −80°C for subsequent DNA and RNA extraction or (ii) homogenised in TET buffer and flash-frozen for subsequent nutrient quantification (see below). From day 10 onwards, when all broods consisted of 100% diapausing larvae, most of the collected larvae were no longer frozen immediately but maintained at 6°C and constant darkness for prolonged diapause. Over a 6-month timecourse, batches of 10–12 larvae were collected and frozen every month for DNA/RNA extraction and nutrient quantification. Survival of the diapausing larvae was measured after 5 and 6 months of cold exposure, in 34 and 41 sibling groups, respectively. Survival was determined based on two criteria: (i) healthy appearance, i.e., absence of necrosis, and (ii) larvae wriggle when poked. In addition, several batches of 10 diapausing larvae (each batch being a sibling group) were transferred back to 25°C and long photoperiod every month in order to terminate diapause and resume development. The emerging adults were sorted into separate pools according to gender and immediately frozen at −80°C for subsequent DNA extraction. The adult sex ratio was strongly biassed towards females, which is typical for *Nasonia*. As it is not possible to determine the gender of larvae, larval pools can be considered to contain both sexes, but with a similarly strong bias towards females.

### In vitro rearing of axenic larvae

Axenic (i.e., germ-free) non-diapausing and diapausing larvae were produced using the same experimental setup as above in combination with the in vitro germ-free rearing method established for *Nasonia* [47, 48]. *Nasonia* embryos were manually collected from parasitized fly hosts after 24h, placed into transwell inserts with permeable 3μm-pore membranes (Corning) and surface-sterilised by rinsing twice with 10% bleach, once with 70% ethanol and three times with sterile water. The transwell inserts were then placed into 24-well plates containing 200 μl of sterile *Nasonia* Rearing Medium [47, 48]. The plates were maintained at 25°C under laminar flow, and the rearing medium was renewed daily until completion of larval development. The axenic status of the larvae was verified in two ways: (i) Absence of bacterial growth in the *Nasonia* Rearing Medium, which would manifest itself through a milky colour of the medium, and (ii) plating of randomly chosen larvae homogenised in sterile PBS onto LB agar plates to confirm absence of bacterial growth. Conventionally reared larvae were plated in the same way as positive controls. As for the conventionally reared larvae, non-diapausing larvae were obtained from 2- to 4-day-old females and their diapause-destined siblings were obtained from 12- to 26-day-old females, when the switch to all-diapause broods was completed. The majority of the diapausing larvae were transferred to 6°C and constant darkness for up to 6 months of diapause.

### Quantification of total protein and nutrient levels

Groups of 10 live conventionally or axenically reared larvae were collected for each experimental condition (i.e., 7-day-old non-diapausing larvae, 7-day-old diapausing larvae, larvae after 1 month, 3 months and 6 months of diapause). Each batch of 10 larvae was weighed to the nearest mg and then crushed in 140 μl ice-cold TET buffer [10 mM **T**ris-HCl (pH 8), 1 mM **E**DTA, 0.1 % (v/v) **T**riton X-100] using sterile pestles. The homogenate was centrifuged for 1 min at 17,000*g* to spin down cell debris. Forty microliters of the supernatant was flash-frozen in liquid nitrogen and stored at −80°C for subsequent protein quantification. The remaining 100 μl of the supernatant was incubated at 72°C for 20 min to inactivate endogenous enzymes prior to freezing in liquid nitrogen and storage at −80°C, as this has been shown to reduce variability between replicates for carbohydrate and lipid quantifications [27].

Subsequently, total protein content was quantified using the colorimetric DC Protein Assay Kit (Bio-Rad) and glucose was quantified using the colorimetric Glucose Assay Kit (Sigma GAGO-20) according to the manufacturer’s instructions. Glycerol and triglycerides were quantified in two steps using the Free Glycerol Reagent for the colorimetric quantification of glycerol (Sigma F6428) and the Triglyceride Reagent (Sigma T2449), which hydrolyses triglycerides into glycerol and fatty acids. Absorbance was measured in triplicates for each sample on a Multiskan FC microplate reader (Thermo Scientific), along with standards and blanks for each assay. Protein and nutrient levels were normalised by the fresh weight of the larvae, in order to correct for size differences between conventionally and axenically reared larvae. Differences in weight, total protein and nutrient levels between conventional and axenic larvae were tested for each experimental condition using the nonparametric Wilcoxon rank sum test. Differences in weight, total protein and nutrient levels over the entire timecourse were tested for conventional and axenic larvae using the nonparametric Kruskal-Wallis rank sum test, followed by Dunn’s post hoc test for multiple comparisons with Benjamini-Hochberg correction (R package “agricolae”).

### DNA and RNA extraction

All insects were surface-sterilised prior to nucleic acid extraction by rinsing once in 10% bleach and twice in sterile water. DNA and RNA were extracted from the same samples, i.e., 9–21 pools of larvae and 4–8 pools of adults for each experimental condition (Supplementary Table 3) using the AllPrep DNA/RNA Mini Kit (QIAGEN). Pools generally consisted of 10–12 larvae, except for non-diapausing larvae were clutch sizes were occasionally lower, resulting in 5–10 larvae per pool. As the number of adults emerging after diapause termination was highly variable, adult pools consisted of 2–6 individuals per pool. Extraction controls were included in each extraction batch. DNA and RNA yields were quantified using the Qubit dsDNA Broad Range Assay Kit and Qubit RNA Broad Range Assay Kit (Invitrogen), respectively.

### Quantification of bacterial titres

In order to quantify the bacterial titre in each sample, the bacterial 16S rRNA gene was amplified using the universal bacterial primers 27F (5′-AGAGTTTGATCCTGGCTCA-3′) and 338R (5′-CTGCTGCCTCCCGTAGGAGT-3′). The single-copy gene coding for the ribosomal protein S6 kinase (NvS6K) was also quantified to normalise bacterial titre per host cell (NvS6K-F: 5′-GGCATTATCTACAGAGATTTGAAACCAG-3′and NvS6K-R: 5′-CAAAGCTATATGACCTTCTGTATCA-3′ [64]). Standard curves were established for both genes based on serial dilutions of purified larger PCR products of known concentrations to calculate the copy number of each gene. Quantitative PCR was performed in a StepOnePlus Real-Time PCR system (Applied Biosystems) using the GoTag qPCR Master Mix (Promega). Twenty microliters of reactions contained 10 μl of 2X GoTaq qPCR Master Mix, 6 μl of sterile water, 0.5 μM of each primer and 2 μl of template DNA. PCR cycles consisted of an initial activation at 95°C for 10 min, followed by 40 cycles of 95°C for 15 s, 60°C for 30 s and 72°C for 30 s. All samples were run in duplicates and a melt curve was included at the end of each run to verify the specificity of the PCR products.

Considering that diploid female *Nasonia* have twice the number of NvS6K copies than haploid males, the NvS6K copy number of adult female samples was divided by two to accurately determine the number of host cells. Differences in bacterial titre/host cell between all experimental conditions were tested using the nonparametric Kruskal-Wallis rank sum test, followed by Dunn’s post hoc test for multiple comparisons with Benjamini-Hochberg correction. Additionally, bacterial titres in adults after diapause termination were compared to those of adults developed from non-diapausing larvae using pairwise Wilcoxon rank sum tests.

### 16S rRNA gene amplicon sequencing

The variable regions V3–V4 of the bacterial 16S rRNA gene were amplified using a seminested PCR, since the commonly used primers 338F and 806R were found to frequently amplify the host’s 18S rRNA gene. To circumvent this issue, we first amplified the V1–V4 regions using the primers 27F and 806R. The 25 μl reactions contained 12.5 μl of Q5 High Fidelity Master Mix (New England Biolabs), 7 μl of sterile water, 0.5 μM of each primer and 4 μl of template DNA. PCR cycles consisted of an initial activation at 98°C for 30 s, followed by 25 cycles of 98°C for 30 s, 60°C for 30 s, 72°C for 30 s and a final extension at 72°C for 10 min. Two microliters of this first reaction was used as template in a second PCR with dual-indexed V3-V4 primers 338F-mod (5′-TACGGRRSGCAGCAGTRRGGAAT-3′) and 806R (5′-GGACTACHVGGGTWTCTAAT-3′). Primer constructs consisted of Illumina adapters, 8-bp barcodes, pad and linker sequences [65]. PCR conditions were the same as for the first amplification, but with only 12 cycles. The seminested PCR approach was validated using mock bacterial communities and *N. vitripennis* samples amplified using both direct and seminested PCR (Supplementary Fig. S5). Each sample was amplified in triplicates, and the three PCR products of each sample were pooled and purified using AMPure XP magnetic beads (Agencourt). Purified PCR products were quantified using the Qbit dsDNA HS Assay Kit (Life Technologies). Extraction controls and PCR negative controls were included but did not yield detectable amounts of DNA. Equimolar amounts of all samples were combined in a final pool. The pooled library was sequenced on an Illumina MiSeq using 2×250 bp paired-end chemistry by the Beijing Genomics Institute (China).

### Microbiome analysis

Raw Illumina reads were processed using QIIME 1.9 [66]. Read pairs were joined and only reads with an average quality ≥30 were retained. No barcode errors or ambiguous bases were allowed. Chimeric reads were removed using usearch v11.0.667 [67]. The remaining reads were clustered into Operational Taxonomic Units (OTUs) at 97% similarity using uclust [67]. Representative sequences from each OTU were aligned against the Silva reference alignment release 132 [68] using PyNAST [69] and identified using the RDP Classifier [70]. Rare OTUs represented by < 5 sequences were not included in subsequent analyses. OTU richness and diversity in each sample were determined using the nonparametric species richness estimator Chao1 and the Shannon Index of diversity after rarefaction to an even sampling depth of 5000 reads for larvae samples and 4000 reads for adult samples (Table S1). Alpha diversity indices were compared between experimental conditions using two-sample *t* tests after 1000 Monte-Carlo permutations as implemented in QIIME. Betadiversity was investigated using Principal Coordinates Analysis (PCoA) based on unweighted UniFrac distances [71]. To test for significant differences in microbiome composition between experimental conditions, we used adonis and anosim analyses implemented in QIIME based on unweighted unifrac distances and 10,000 permutations. Correlations between microbial community structure and environmental factors (i.e., host nutrient levels and temperature) were investigated by fitting the environmental variables onto Nonmetric Multidimensional Scaling ordination scores using the EnvFit function (R package “vegan”). Differentially abundant bacterial genera in diapausing vs. non-diapausing wasps were determined using the edgeR package for differential gene expression analysis [72]. Abundance was based on log CPM (Counts Per Million) values. Only genera present in at least four samples were included in the analysis and genera were considered differentially abundant with a log2-fold change ≥ 1 and an FDR-adjusted *p* value ≤ 0.05.

### RNA sequencing

RNAseq libraries were prepared for three biological replicates (pools of 10 sibling larvae) per experimental condition (i.e., 7-day-old non-diapausing larvae, 7-day-old early diapause larvae, larvae after 1 month and 6 months of diapause). Intermediate time points could not be included due to insufficient RNA yields from 2 to 5 month diapause samples. RNA quality was verified using TapeStation RNA Screen Tapes (Agilent). Automated RNAseq library preparation was performed using PrepX reagent kits (Wafergen) on the Apollo 324 liquid handling system (Takara) at the Bauer Core Facility at Harvard University. Starting with 1 μg total RNA, polyA-selection was performed using the PrepX PolyA mRNA Isolation Kit and cDNA libraries were prepared using the PrepX RNA-Seq for Illumina Library Kit and Superscript III reverse transcriptase (Invitrogen). The resulting cDNA libraries were PCR-amplified for 15 cycles with Illumina Index primers using the LongAmp Taq Master Mix (New England Biolabs). PCR products were purified using Aline magnetic beads (Aline Biosciences). Library quantity and quality was verified using the Qubit DNA High Sensitivity Assay Kit (Invitrogen), TapeStation D1000 DNA Screen Tapes (Agilent) and quantitative PCR using the KAPA SYBR Fast ABI kit (KAPA Biosystems). Single-end sequencing (100 bp) was performed on an Illumina Hiseq 2500 at the Bauer Core Facility at Harvard University.

### RNASeq data analysis

RNAseq raw reads were quality-trimmed using Trimmomatic [73]. Illumina adapters were removed using ILLUMINACLIP, reads were end-trimmed when average phred-scores dropped < 30 over a sliding window of four bases and the first five bases showing non-random base composition were cut off from the beginning of each read. Only reads of at least 94 bases after these procedures were retained for subsequent analyses. This resulted in 4–6 million high-quality reads per library. The reads from each library were mapped against the *N. vitripennis* genome assembly 2.1 (GCF_000002325.3 [74]) using HISAT2 [75, 76] and gene expression levels were quantified using HTSeq [77]. Differential expression analysis was performed using edgeR [72], and genes were considered differentially expressed with a log_2_-fold change ≥ 1 and a Benjamini-Hochberg FDR-corrected *p* value < 0.05. Differentially expressed genes were assigned to KEGG pathways using the KEGG Automatic Annotation Server KAAS (https://www.genome.jp/tools/kaas/).

## Supplementary Information

**Additional file 1:** **Supplementary Fig. S1**. Larval microbiome composition in each replicate sample. Each sample represents a pool of 5-12 sibling larvae. See Supplementary Table 1 for detailed information on each sample. The most abundant bacterial genera are represented in the legend.

**Additional file 2:** **Supplementary Fig. S2**. Bacterial richness and diversity in larvae and adults based on the species richness estimator Chao 1 and the Shannon Index of diversity. Alphadiversity indices were compared between experimental conditions using pairwise t-tests after 1000 Monte-Carlo permutations.

**Additional file 3:** **Supplementary Fig. S3**. Abundance of 52 genera in each replicate larvae sample. Each genus was differentially abundant in at least one diapause condition compared to non-diapausing larvae. Rows are ordered based on abundance patterns using hierarchical clustering. A summarized version showing the mean abundances of these genera per experimental condition is shown in Fig. 4.

**Additional file 4:** **Supplementary Fig. S4.** Adult microbiome composition in each replicate sample. Each sample represents a pool of 2-6 siblings. See Supplementary Table 1 for detailed information on each sample. The most abundant bacterial genera are represented in the legend.

**Additional file 5:** **Supplementary Fig. S5.** Validation of the seminested PCR approach. (A) Direct vs. seminested amplification of the V3-V4 region using a commercially available mock bacterial community containing 15 bacterial genera (Cat. No. HM-782D, Bei Resources). Both amplifications captured all 15 genera and did not differ in bacterial species richness and diversity. (B) The seminested approach captured a higher bacterial species richness in *N. vitripennis* larvae compared to direct amplification. Differences in alphadiversity were determined using t-tests after 1000 Monte-Carlo permutations, on an even sampling depth of 1200 reads/sample (mock community) and 3000 reads/sample (*Nasonia* larvae).

**Additional file 6:** **Supplementary Table 1**. Weight, protein and nutrient quantification dataset from conventional and axenic larvae.

**Additional file 7:** **Supplementary Table 2.** qPCR quantification of the *Nasonia* NvS6k gene and the bacterial 16S rRNA gene in larvae and adults.

**Additional file 8:** **Supplementary Table 3**. Overview of the 138 amplicon pools used in this work, providing sequence yield, OTU number and estimates of bacterial richness and diversity for each sample.

**Additional file 9:** **Supplementary Table 4.** Excel file containing the OTU tables used for microbiome analyses, counts of all bacterial genera by experimental condition, and log CPM values of the differentially abundant genera.

**Additional file 10:** **Supplementary Table 5**. Sequence yield and mapping rate for RNAseq samples.

## Data Availability

The 16S rRNA amplicon sequencing and RNAseq reads produced for this study are available in the NCBI Sequence Read Archive under project numbers PRJNA611497 (amplicon sequencing) and PRJNA611533 (RNAseq). All other datasets generated during this study are provided as supplementary material (Supplementary Tables 1 and 2).

## References

[1] Denlinger DL (2002). Regulation of diapause. Annu Rev Entomol.

[2] Saunders DS (2000). Larval diapause duration and fat metabolism in three geographical strains of the blow fly, *Calliphora vicina*. J Insect Physiol.

[3] Paolucci S, van de Zande L, Beukeboom LW (2013). Adaptive latitudinal cline of photoperiodic diapause induction in the parasitoid *Nasonia vitripennis* in Europe. J Evol Biol.

[4] Urbanski J, Mogi M, O'Donnell D, DeCotiis M, Toma T, Armbruster P (2012). Rapid adaptive evolution of photoperiodic response during invasion and range expansion across a climatic gradient. Am Nat.

[5] Poelchau MF, Reynolds JA, Elsik CG, Denlinger DL, Armbruster PA (2013). Deep sequencing reveals complex mechanisms of diapause preparation in the invasive mosquito, *Aedes albopictus*. Proc Biol Sci.

[6] Sim C, Denlinger DL (2013). Insulin signaling and the regulation of insect diapause. Front Physiol.

[7] Reynolds JA, Poelchau MF, Rahman Z, Armbruster PA, Denlinger DL (2012). Transcript profiling reveals mechanisms for lipid conservation during diapause in the mosquito, *Aedes albopictus*. J Insect Physiol.

[8] Hahn DA, Denlinger DL (2007). Meeting the energetic demands of insect diapause: nutrient storage and utilization. J Insect Physiol.

[9] Denlinger DL, Shukla M, Faustini DL (1984). Juvenile hormone involvement in pupal diapause of the flesh fly *Sarcophaga crassipalpis*: regulation of infradian cycles of O_2_ consumption. J Exp Biol.

[10] Kostal V (2006). Eco-physiological phases of insect diapause. J Insect Physiol.

[11] Yamashita O (1996). Diapause hormone of the silkworm, *Bombyx mori*: structure, gene expression and function. J Insect Physiol.

[12] Kucerova L, Kubrak OI, Bengtsson JM, Strnad H, Nylin S, Theopold U, et al. Slowed aging during reproductive dormancy is reflected in genome-wide transcriptome changes in *Drosophila melanogaster*. BMC Genomics. 2016;17(1):50. 10.1186/s12864-016-2383-1. 10.1186/s12864-016-2383-1 PMC4711038 26758761

[13] Bajgar A, Jindra M, Dolezel D (2013). Autonomous regulation of the insect gut by circadian genes acting downstream of juvenile hormone signaling. Proc Natl Acad Sci U S A.

[14] Poelchau MF, Reynolds JA, Elsik CG, Denlinger DL, Armbruster PA (2013). RNA-Seq reveals early distinctions and late convergence of gene expression between diapause and quiescence in the Asian tiger mosquito, *Aedes albopictus*. J Exp Biol.

[15] Liu W, Li Y, Guo S, Yin H, Lei CL, Wang XP (2016). Association between gut microbiota and diapause preparation in the cabbage beetle: a new perspective for studying insect diapause. Sci Rep.

[16] Mushegian AA, Tougeron K (2019). Animal-microbe interactions in the context of diapause. Biol Bull.

[17] Medina V, Sardoy PM, Soria M, Vay CA, Gutkind GO, Zavala JA (2018). Characterized non-transient microbiota from stinkbug (*Nezara viridula*) midgut deactivates soybean chemical defenses. PLoS One.

[18] Ferguson LV, Dhakal P, Lebenzon JE, Heinrichs DE, Bucking C, Sinclair BJ (2018). Seasonal shifts in the insect gut microbiome are concurrent with changes in cold tolerance and immunity. Funct Ecol.

[19] Dill-McFarland KA, Neil KL, Zeng A, Sprenger RJ, Kurtz CC, Suen G, et al. Hibernation alters the diversity and composition of mucosa-associated bacteria while enhancing antimicrobial defence in the gut of 13-lined ground squirrels. Mol Ecol. 2014;23(18):4658–69. 10.1111/mec.12884. 10.1111/mec.12884 25130694

[20] Carey HV, Walters WA, Knight R (2013). Seasonal restructuring of the ground squirrel gut microbiota over the annual hibernation cycle. Am J Physiol Regul Integr Comp Physiol.

[21] Stevenson TJ, Duddleston KN, Buck CL (2014). Effects of season and host physiological state on the diversity, density, and activity of the arctic ground squirrel cecal microbiota. Appl Environ Microbiol.

[22] Sommer F, Stahlman M, Ilkayeva O, Arnemo JM, Kindberg J, Josefsson J, et al. The gut microbiota modulates energy metabolism in the hibernating brown bear *Ursus arctos*. Cell Rep. 2016;14(7):1655–61. 10.1016/j.celrep.2016.01.026. 10.1016/j.celrep.2016.01.026 26854221

[23] van Opstal EJ, Bordenstein SR (2019). Phylosymbiosis impacts adaptive traits in *nasonia* wasps. mBio.

[24] Coon KL, Brown MR, Strand MR (2016). Gut bacteria differentially affect egg production in the anautogenous mosquito *Aedes aegypti* and facultatively autogenous mosquito *Aedes atropalpus* (Diptera: Culicidae). Parasit Vectors.

[25] Shin SC, Kim SH, You H, Kim B, Kim AC, Lee KA, et al. *Drosophila* microbiome modulates host developmental and metabolic homeostasis via insulin signaling. Science. 2011;334(6056):670–4. 10.1126/science.1212782. 10.1126/science.1212782 22053049

[26] Storelli G, Defaye A, Erkosar B, Hols P, Royet J, Leulier F (2011). *Lactobacillus plantarum* promotes *Drosophila* systemic growth by modulating hormonal signals through TOR-dependent nutrient sensing. Cell Metabolism.

[27] Newell PD, Douglas AE (2014). Interspecies interactions determine the impact of the gut microbiota on nutrient allocation in *Drosophila melanogaster*. Appl Environ Microbiol.

[28] Wong AC, Dobson AJ, Douglas AE (2014). Gut microbiota dictates the metabolic response of *Drosophila* to diet. J Exp Biol.

[29] Zheng H, Powell JE, Steele MI, Dietrich C, Moran NA (2017). Honeybee gut microbiota promotes host weight gain via bacterial metabolism and hormonal signaling. Proc Natl Acad Sci U S A.

[30] Ellers J, van Alphen JJM (2002). A trade-off between diapause duration and fitness in female parasitoids. Ecol Entomol.

[31] Urbanski JM, Benoit JB, Michaud MR, Denlinger DL, Armbruster P (2010). The molecular physiology of increased egg desiccation resistance during diapause in the invasive mosquito, *Aedes albopictus*. Proc Biol Sci.

[32] Lee J, Kim CH, Jang HA, Kim JK, Kotaki T, Shinoda T, et al. *Burkholderia* gut symbiont modulates titer of specific juvenile hormone in the bean bug *Riptortus pedestris*. Dev Comp Immunoli. 2019;99:103399. 10.1016/j.dci.2019.103399. 10.1016/j.dci.2019.103399 31195052

[33] Sicard M, Dittmer J, Greve P, Bouchon D, Braquart-Varnier C (2014). A host as an ecosystem: *Wolbachia* coping with environmental constraints. Environ Microbiol.

[34] Perrot-Minnot MJ, Guo LR, Werren JH (1996). Single and double infections with *Wolbachia* in the parasitic wasp *Nasonia vitripennis*: effects on compatibility. Genetics.

[35] Dittmer J, van Opstal EJ, Shropshire JD, Bordenstein SR, Hurst GD, Brucker RM (2016). Disentangling a holobiont - recent advances and perspectives in *Nasonia* wasps. Front Microbiol.

[36] Paolucci S, Salis L, Vermeulen CJ, Beukeboom LW, van de Zande L (2016). QTL analysis of the photoperiodic response and clinal distribution of *period* alleles in *Nasonia vitripennis*. Mol Ecol.

[37] Bertossa RC, van de Zande L, Beukeboom LW, Beersma DG (2014). Phylogeny and oscillating expression of *period* and *cryptochrome* in short and long photoperiods suggest a conserved function in *Nasonia vitripennis*. Chronobiol Int.

[38] Pegoraro M, Bafna A, Davies NJ, Shuker DM, Tauber E (2016). DNA methylation changes induced by long and short photoperiods in *Nasonia*. Genome Res.

[39] Mukai A, Goto SG (2016). The clock gene *period* is essential for the photoperiodic response in the jewel wasp *Nasonia vitripennis* (Hymenoptera: Pteromalidae). Appl Entomol Zool.

[40] Wang GH, Berdy BM, Velasquez O, Jovanovic N, Alkhalifa S, Minbiole KPC, et al. Changes in microbiome confer multigenerational host resistance after sub-toxic pesticide exposure. Cell Host Microbe. 2020;27(2):213–24. 10.1016/j.chom.2020.01.009. 10.1016/j.chom.2020.01.009 32023487

[41] Brooks AW, Kohl KD, Brucker RM, van Opstal EJ, Bordenstein SR (2016). Phylosymbiosis: Relationships and functional effects of microbial communities across host evolutionary history. PLoS Biol.

[42] Brucker RM, Bordenstein SR (2013). The hologenomic basis of speciation: gut bacteria cause hybrid lethality in the genus *Nasonia*. Science.

[43] Brucker RM, Bordenstein SR (2012). The roles of host evolutionary relationships (genus: *Nasonia*) and development in structuring microbial communities. Evolution.

[44] Saunders DS (1966). Larval diapause of maternal origin - II. The effect of photoperiod and temperature on *Nasonia vitripennis*. J Insect Physiol.

[45] Saunders DS (1965). Larval diapause of maternal origin: induction of diapause in *Nasonia vitripennis* (Walk.) (Hymenoptera: Pteromalidae). J Exp Biol.

[46] Shimizu Y, Mukai A, Goto SG (2018). Cell cycle arrest in the jewel wasp *Nasonia vitripennis* in larval diapause. J Insect Physiol.

[47] Brucker RM, Bordenstein SR (2012). *In vitro* cultivation of the Hymenoptera genetic model, *Nasonia*. PLoS One.

[48] Shropshire JD, van Opstal EJ, Bordenstein SR (2016). An optimized approach to germ-free rearing in the jewel wasp *Nasonia*. PeerJ.

[49] Wolschin F, Gadau J (2009). Deciphering proteomic signatures of early diapause in *Nasonia*. PLoS One.

[50] Huang X, Poelchau MF, Armbruster PA (2015). Global transcriptional dynamics of diapause induction in non-blood-fed and blood-fed *Aedes albopictus*. PLoS Negl Trop Dis.

[51] Hao YJ, Zhang YJ, Si FL, Fu DY, He ZB, Chen B (2016). Insight into the possible mechanism of the summer diapause of *Delia antiqua* (Diptera: Anthomyiidae) through digital gene expression analysis. Insect Science.

[52] Rawat SR, Mannisto MK, Starovoytov V, Goodwin L, Nolan M, Hauser L, et al. Complete genome sequence of *Terriglobus saanensis* type strain SP1PR4(T), an *Acidobacteria* from tundra soil. Standards in Genomic Sciences. 2012;7(1):59–69. 10.4056/sigs.3036810. 10.4056/sigs.3036810 PMC3570800 23450133

[53] Eichorst SA, Breznak JA, Schmidt TM (2007). Isolation and characterization of soil bacteria that define *Terriglobus* gen. nov., in the phylum *Acidobacteria*. Appl Environ Microbiol.

[54] Franzenburg S, Walter J, Kunzel S, Wang J, Baines JF, Bosch TC, et al. Distinct antimicrobial peptide expression determines host species-specific bacterial associations. Proc Natl Acad Sci U S A. 2013;110(39):E3730–8. 10.1073/pnas.1304960110. 10.1073/pnas.1304960110 PMC3785777 24003149

[55] Lee KA, Kim SH, Kim EK, Ha EM, You H, Kim B, et al. Bacterial-derived uracil as a modulator of mucosal immunity and gut-microbe homeostasis in *Drosophila*. Cell. 2013;153(4):797–811. 10.1016/j.cell.2013.04.009. 10.1016/j.cell.2013.04.009 23663779

[56] Flannagan RD, Tammariello SP, Joplin KH, Cikra-Ireland RA, Yocum GD, Denlinger DL (1998). Diapause-specific gene expression in pupae of the flesh fly *Sarcophaga crassipalpis*. Proc Natl Acad Sci U S A.

[57] Rivers DB, Lee RE, Denlinger DL (2000). Cold hardiness of the fly pupal parasitoid *Nasonia vitripennis* is enhanced by its host *Sarcophaga crassipalpis*. J Insect Physiol.

[58] Gherna RL, Werren JH, Weisburg W, Cote R, Woese CR, Mandelco L, et al. *Arsenophonus nasoniae* gen. nov., sp. nov. the causative agent of the son-killer trait in the parasitic wasp *Nasonia vitripennis*. Int J Syst Bacteriol. 1991;41(4):563–5. 10.1099/00207713-41-4-563.

[59] Nadal-Jimenez P, Griffin JS, Davies L, Frost CL, Marcello M, Hurst GDD (2019). Genetic manipulation allows in vivo tracking of the life cycle of the son-killer symbiont, *Arsenophonus nasoniae*, and reveals patterns of host invasion, tropism and pathology. Environ Microbiol.

[60] Darby AC, Choi JH, Wilkes T, Hughes MA, Werren JH, Hurst GD, et al. Characteristics of the genome of *Arsenophonus nasoniae*, son-killer bacterium of the wasp *Nasonia*. Insect Mol Biol. 2010;19(Suppl 1):75–89. 10.1111/j.1365-2583.2009.00950.x. 10.1111/j.1365-2583.2009.00950.x 20167019

[61] Wilkes TE, Darby AC, Choi JH, Colbourne JK, Werren JH, Hurst GD (2010). The draft genome sequence of *Arsenophonus nasoniae*, son-killer bacterium of *Nasonia vitripennis*, reveals genes associated with virulence and symbiosis. Insect Mol Biol.

[62] Manzano-Marin A, Oceguera-Figueroa A, Latorre A, Jimenez-Garcia LF, Moya A (2015). Solving a bloody mess: B-vitamin independent metabolic convergence among gammaproteobacterial obligate endosymbionts from blood-feeding arthropods and the leech *Haementeria officinalis*. Genome Biol Evol.

[63] Yoshiyama M, Kimura K (2009). Bacteria in the gut of Japanese honeybee, *Apis cerana japonica*, and their antagonistic effect against *Paenibacillus larvae*, the causal agent of American foulbrood. J Invertebr Pathol.

[64] Funkhouser-Jones LJ, van Opstal EJ, Sharma A, Bordenstein SR (2018). The maternal effect gene *Wds* controls *Wolbachia* titer in *Nasonia*. Curr Biol.

[65] Kozich JJ, Westcott SL, Baxter NT, Highlander SK, Schloss PD (2013). Development of a dual-index sequencing strategy and curation pipeline for analyzing amplicon sequence data on the MiSeq Illumina sequencing platform. Appl Environ Microbiol.

[66] Caporaso JG, Kuczynski J, Stombaugh J, Bittinger K, Bushman FD, Costello EK, et al. QIIME allows analysis of high-throughput community sequencing data. Nat Methods. 2010;7(5):335–6. 10.1038/nmeth.f.303. 10.1038/nmeth.f.303 PMC3156573 20383131

[67] Edgar RC (2010). Search and clustering orders of magnitude faster than BLAST. Bioinformatics.

[68] Quast C, Pruesse E, Yilmaz P, Gerken J, Schweer T, Yarza P, et al. The SILVA ribosomal RNA gene database project: improved data processing and web-based tools. Nucleic Acids Res. 2013;41(Database issue):D590–6. 10.1093/nar/gks1219. 10.1093/nar/gks1219 PMC3531112 23193283

[69] Caporaso JG, Bittinger K, Bushman FD, DeSantis TZ, Andersen GL, Knight R (2010). PyNAST: a flexible tool for aligning sequences to a template alignment. Bioinformatics.

[70] Wang Q, Garrity GM, Tiedje JM, Cole JR (2007). Naive Bayesian classifier for rapid assignment of rRNA sequences into the new bacterial taxonomy. Appl Environ Microbiol.

[71] Lozupone C, Knight R (2005). UniFrac: a new phylogenetic method for comparing microbial communities. Appl Environ Microbiol.

[72] Robinson MD, McCarthy DJ, Smyth GK (2010). edgeR: a Bioconductor package for differential expression analysis of digital gene expression data. Bioinformatics.

[73] Bolger AM, Lohse M, Usadel B (2014). Trimmomatic: a flexible trimmer for Illumina sequence data. Bioinformatics.

[74] Werren JH, Richards S, Desjardins CA, Niehuis O, Gadau J, Colbourne JK, et al. Functional and evolutionary insights from the genomes of three parasitoid *Nasonia species*. Science. 2010;327(5963):343–8. 10.1126/science.1178028. 10.1126/science.1178028 PMC2849982 20075255

[75] Pertea M, Kim D, Pertea GM, Leek JT, Salzberg SL (2016). Transcript-level expression analysis of RNA-seq experiments with HISAT, StringTie and Ballgown. Nat Protoc.

[76] Kim D, Langmead B, Salzberg SL (2015). HISAT: a fast spliced aligner with low memory requirements. Nat Methods.

[77] Anders S, Pyl PT, Huber W (2015). HTSeq--a Python framework to work with high-throughput sequencing data. Bioinformatics.

