# Supplementary material for: When your host shuts down: larval diapause impacts host-microbiome interactions in Nasonia vitripennis
Source: Microbiome. 2021 Apr 9;9:85. doi: 10.1186/s40168-021-01037-6 (PMC8035746; doi:10.1186/s40168-021-01037-6)
Supplement: Supplementary file 3 — Additional file 2: Supplementary Fig. S2. Bacterial richness and diversity in larvae and adults based on the species richness estimator Chao 1 and the Shannon Index of diversity. Alphadiversity indices were compared between experimental conditions using pairwise t-tests after 1000 Monte-Carlo permutations. [file 40168_2021_1037_MOESM3_ESM.pdf]

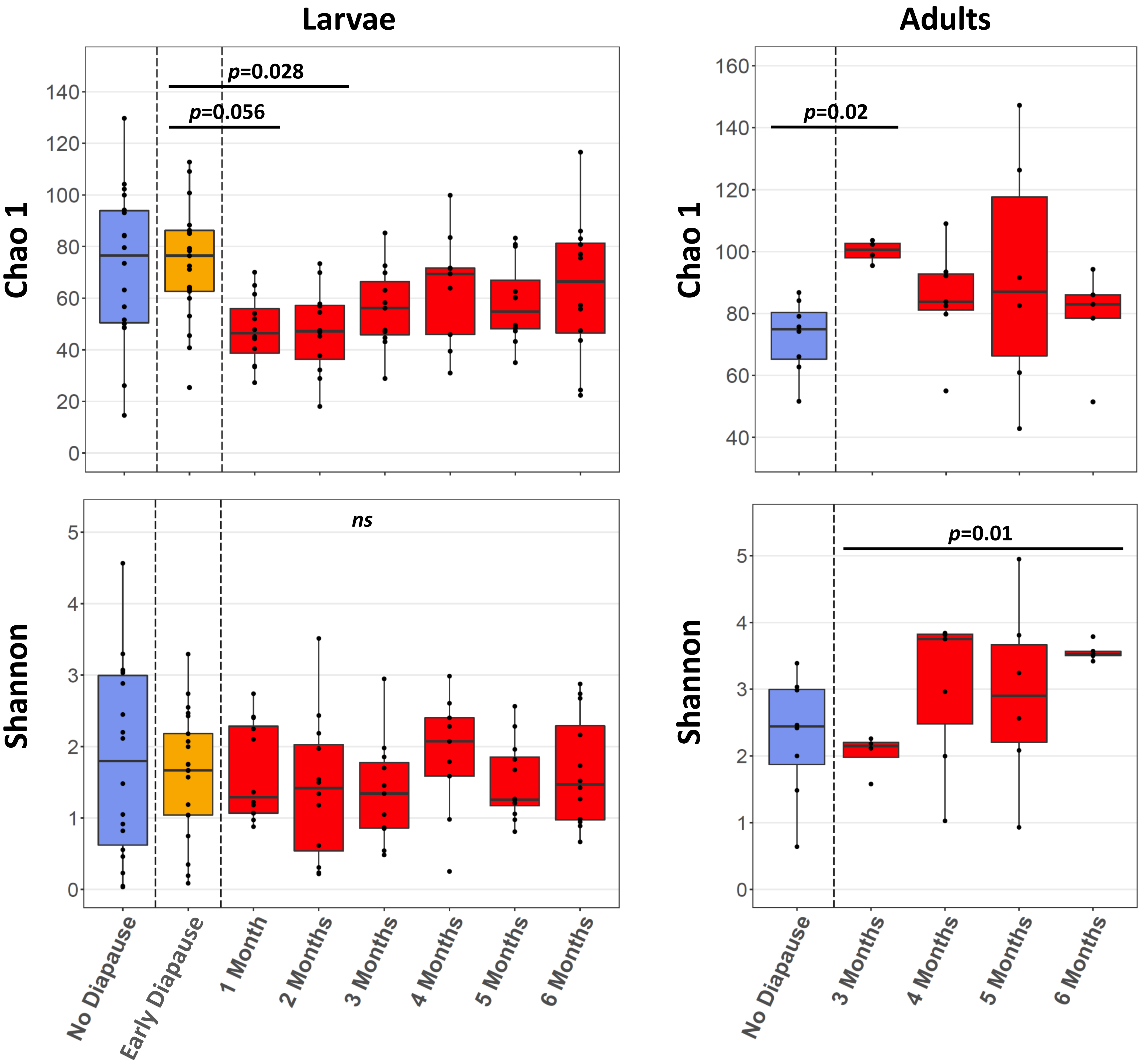

Supplementary Figure S2. Bacterial richness and diversity in larvae and adults based on the species richness estimator Chao 1 and the Shannon Index of diversity. Alphadiversity indices were compared between experimental conditions using pairwise t-tests after 1000 Monte-Carlo permutations.
