# Supplementary material for: When your host shuts down: larval diapause impacts host-microbiome interactions in Nasonia vitripennis
Source: Microbiome. 2021 Apr 9;9:85. doi: 10.1186/s40168-021-01037-6 (PMC8035746; doi:10.1186/s40168-021-01037-6)
Supplement: Supplementary file 4 — Additional file 3: Supplementary Fig. S3. Abundance of 52 genera in each replicate larvae sample. Each genus was differentially abundant in at least one diapause condition compared to non-diapausing larvae. Rows are ordered based on abundance patterns using hierarchical clustering. A summarized version showing the mean abundances of these genera per experimental condition is shown in Fig. 4. [file 40168_2021_1037_MOESM4_ESM.pdf]

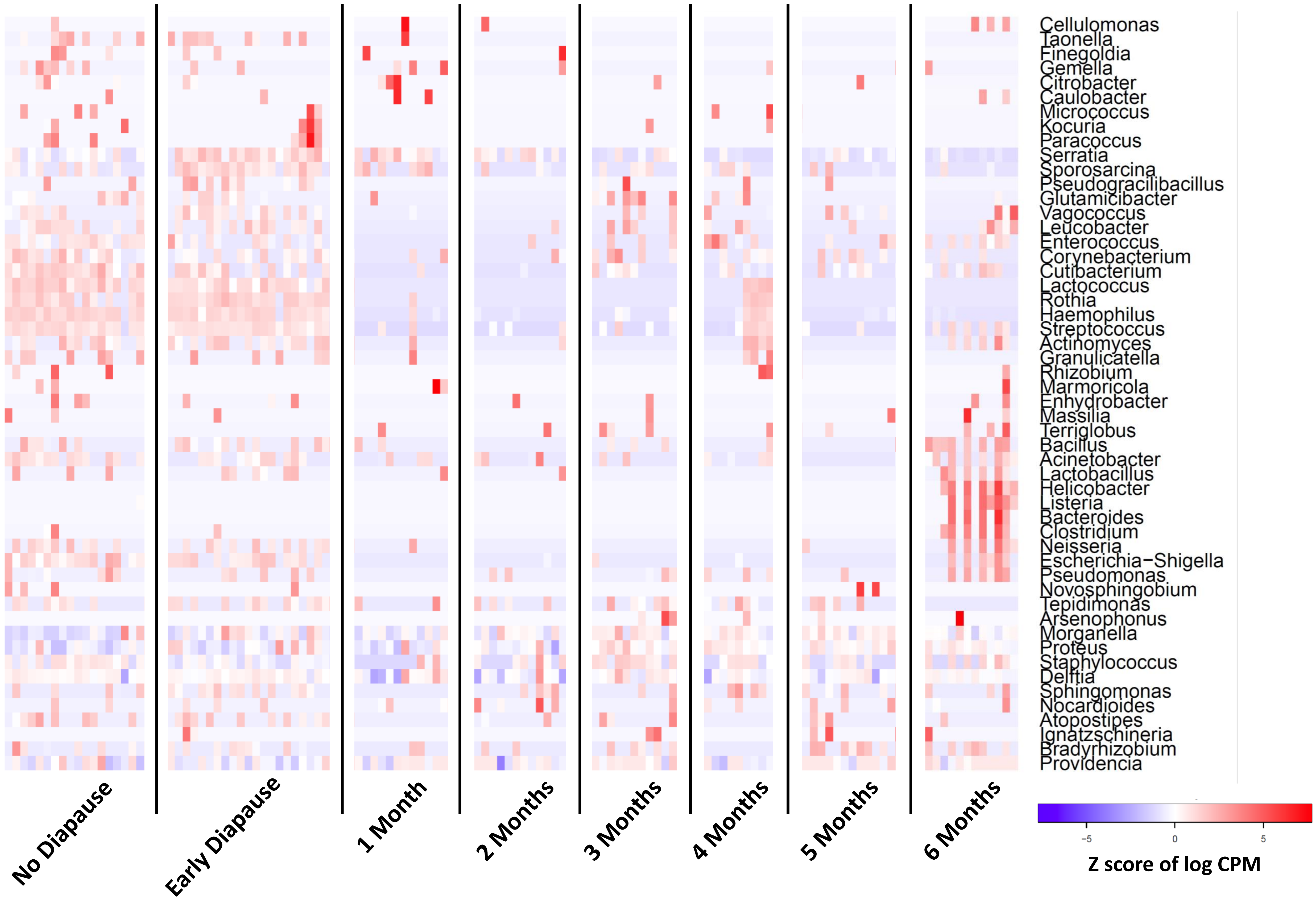


**Supplementary Figure S3.** Abundance of 52 genera in each replicate larvae sample. Each genus was differentially abundant in at least one diapause condition compared to non-diapausing larvae. Rows are ordered based on abundance patters using hierarchical clustering. A summarized version showing the mean abundances of these genera per experimental condition is shown in Fig. 4.
