# Supplementary material for: When your host shuts down: larval diapause impacts host-microbiome interactions in Nasonia vitripennis
Source: Microbiome. 2021 Apr 9;9:85. doi: 10.1186/s40168-021-01037-6 (PMC8035746; doi:10.1186/s40168-021-01037-6)
Supplement: Supplementary file 5 — Additional file 4: Supplementary Fig. S4. Adult microbiome composition in each replicate sample. Each sample represents a pool of 2-6 siblings. See Supplementary Table 1 for detailed information on each sample. The most abundant bacterial genera are represented in the legend. [file 40168_2021_1037_MOESM5_ESM.pdf]

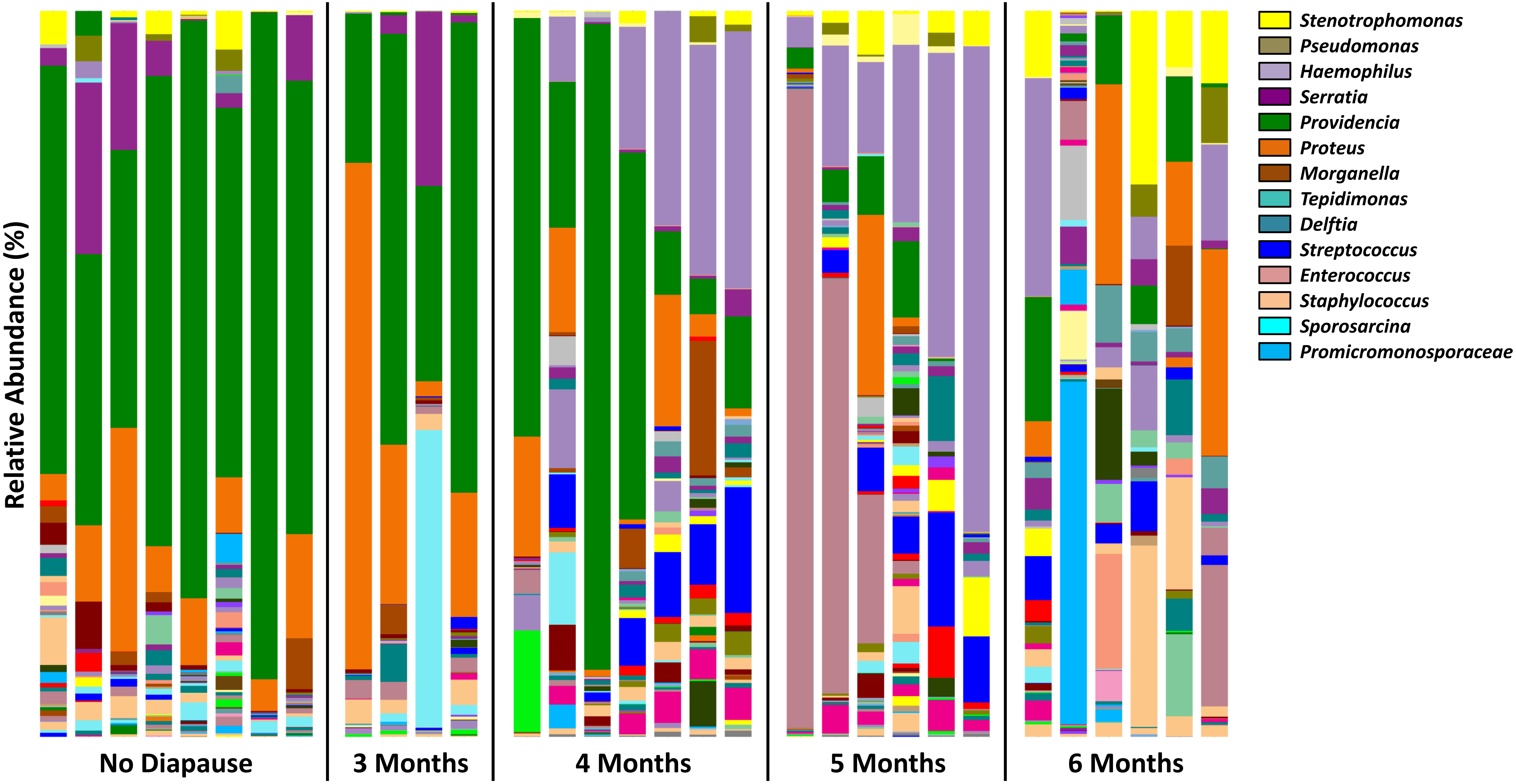

Supplementary Figure S4. Adult microbiome composition in each replicate sample. Each sample represents a pool of 2-6 siblings. See Supplementary Table 1 for detailed information on each sample. The most abundant bacterial genera are represented in the legend.
