# Supplementary material for: When your host shuts down: larval diapause impacts host-microbiome interactions in Nasonia vitripennis
Source: Microbiome. 2021 Apr 9;9:85. doi: 10.1186/s40168-021-01037-6 (PMC8035746; doi:10.1186/s40168-021-01037-6)
Supplement: Supplementary file 6 — Additional file 5: Supplementary Fig. S5. Validation of the seminested PCR approach. (A) Direct vs. seminested amplification of the V3-V4 region using a commercially available mock bacterial community containing 15 bacterial genera (Cat. No. HM-782D, Bei Resources). Both amplifications captured all 15 genera and did not differ in bacterial species richness and diversity. (B) The seminested approach captured a higher bacterial species richness in N. vitripennis larvae compared to direct amplification. Differences in alphadiversity were determined using t-tests after 1000 Monte-Carlo permutations, on an even sampling depth of 1200 reads/sample (mock community) and 3000 reads/sample (Nasonia larvae). [file 40168_2021_1037_MOESM6_ESM.pdf]

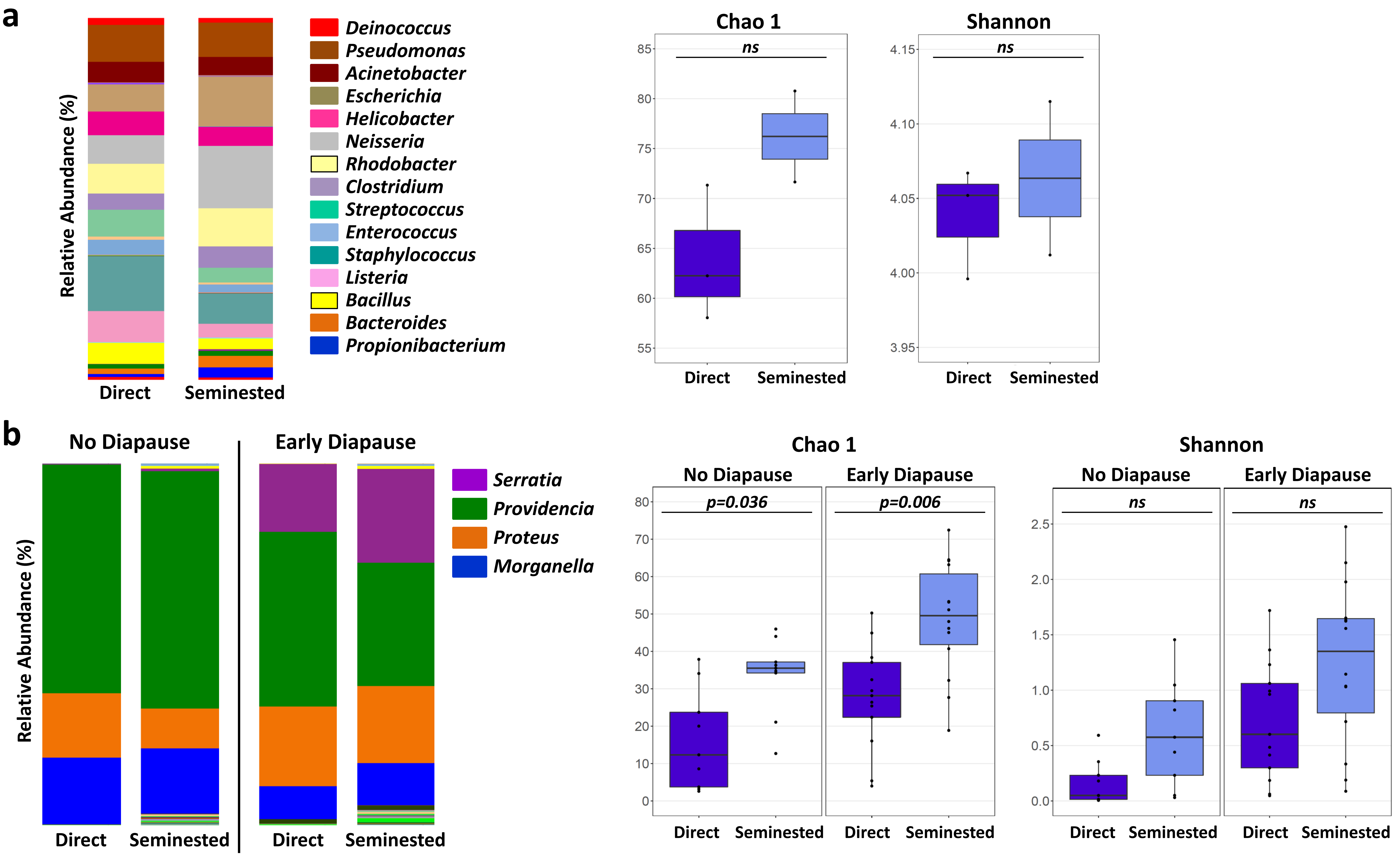


**Supplementary Figure S5.** Validation of the seminested PCR approach. (A) Direct vs. seminested amplification of the V3-V4 region using a commercially available mock bacterial community containing 15 bacterial genera (Cat. No. HM-782D, Bei Resources). Both amplifications captured all 15 genera and did not differ in terms of species richness and diversity. (B) The seminested approach captured a higher bacterial species richness in *N. vitripennis* larvae compared to direct amplification. Differences in alphadiversity were determined using t-tests after 1000 Monte-Carlo permutations, on an even sampling depth of 1200 reads/sample (mock community) and 3000 reads/sample (*Nasonia* larvae).
