# Supplementary material for: When your host shuts down: larval diapause impacts host-microbiome interactions in Nasonia vitripennis
Source: Microbiome. 2021 Apr 9;9:85. doi: 10.1186/s40168-021-01037-6 (PMC8035746; doi:10.1186/s40168-021-01037-6)
Supplement: Supplementary file 9 — Additional file 8: Supplementary Table 3. Overview of the 138 amplicon pools used in this work, providing sequence yield, OTU number and estimates of bacterial richness and diversity for each sample. [file 40168_2021_1037_MOESM9_ESM.doc]

**Supplementary Table 3** Overview of the 138 amplicon pools used in this work

| **Pool No** | **Condition** | **Developmental Stage** | **Mother** | **Age of Mother (Days)** | **No of individuals** | **No of Reads^a^** | **No of OTUs^a^** | **Richness and diversity estimates^b^** | |
| --- | --- | --- | --- | --- | --- | --- | --- | --- | --- |
|  |  |  |  |  |  |  |  | **Chao 1** | **Shannon** |
| 1 | No Diapause | Larvae | V02 | 2 | 8 | 25048 | 88 | 73.503 | 2.113 |
| 2 | No Diapause | Larvae | V02 | 5 | 10 | 32863 | 81 | 63.173 | 1.481 |
| 3 | No Diapause | Larvae | V04 | 3 | 7 | 22451 | 103 | 99.930 | 2.883 |
| 4 | No Diapause | Larvae | V05 | 5 | 10 | 23287 | 98 | 94.189 | 3.296 |
| 5 | No Diapause | Larvae | V07 | 3 | 10 | 37394 | 70 | 51.592 | 1.049 |
| 6 | No Diapause | Larvae | V07 | 4 | 10 | 25623 | 97 | 84.350 | 3.032 |
| 7 | No Diapause | Larvae | V10 | 2 | 7 | 29471 | 152 | 129.715 | 2.199 |
| 8 | No Diapause | Larvae | V10 | 5 | 10 | 28591 | 62 | 56.665 | 0.229 |
| 9 | No Diapause | Larvae | V11 | 2 | 7 | 15109 | 82 | 102.266 | 2.448 |
| 10 | No Diapause | Larvae | V11 | 3 | 10 | 19547 | 84 | 93.101 | 3.072 |
| 11 | No Diapause | Larvae | V13 | 3 | 10 | 32982 | 60 | 50.063 | 0.555 |
| 12 | No Diapause | Larvae | V13 | 4 | 10 | 30119 | 90 | 79.573 | 0.820 |
| 13 | No Diapause | Larvae | V14 | 2 | 10 | 25767 | 38 | 26.092 | 0.047 |
| 14 | No Diapause | Larvae | V14 | 3 | 5 | 17406 | 100 | 104.174 | 4.565 |
| 15 | No Diapause | Larvae | V16 | 2 | 8 | 25530 | 97 | 84.124 | 3.058 |
| 16 | No Diapause | Larvae | V16 | 4 | 10 | 22158 | 19 | 14.575 | 0.033 |
| 17 | No Diapause | Larvae | V17 | 3 | 7 | 22445 | 56 | 48.622 | 0.460 |
| 18 | No Diapause | Larvae | V17 | 4 | 10 | 27375 | 61 | 48.511 | 0.915 |
| 19 | Early Diapause | Larvae | V02 | 10 | 10 | 24532 | 70 | 59.917 | 1.664 |
| 20 | Early Diapause | Larvae | V02 | 13 | 10 | 26707 | 98 | 79.295 | 1.748 |
| 21 | Early Diapause | Larvae | V04 | 11 | 10 | 22295 | 119 | 112.746 | 3.293 |
| 22 | Early Diapause | Larvae | V04 | 13 | 10 | 20419 | 98 | 100.776 | 2.739 |
| 23 | Early Diapause | Larvae | V07 | 11 | 10 | 21759 | 61 | 53.063 | 1.038 |
| 24 | Early Diapause | Larvae | V07 | 13 | 10 | 19722 | 81 | 71.141 | 2.427 |
| 25 | Early Diapause | Larvae | V08 | 11 | 10 | 16835 | 102 | 109.101 | 1.663 |

**Supplementary Table 3 (cont.)** Overview of the 138 amplicon pools used in this work

| **Pool No** | **Condition** | **Developmental Stage** | **Mother** | **Age of Mother (Days)** | **No of individuals** | **No of Reads^a^** | **No of OTUs^a^** | **Richness and diversity estimates^b^** | |
| --- | --- | --- | --- | --- | --- | --- | --- | --- | --- |
|  |  |  |  |  |  |  |  | **Chao 1** | **Shannon** |
| 26 | Early Diapause | Larvae | V08 | 13 | 10 | 24493 | 34 | 25.358 | 0.086 |
| 27 | Early Diapause | Larvae | V09 | 11 | 10 | 20807 | 96 | 86.285 | 2.179 |
| 28 | Early Diapause | Larvae | V09 | 13 | 10 | 25361 | 71 | 64.243 | 1.042 |
| 29 | Early Diapause | Larvae | V10 | 11 | 10 | 23456 | 89 | 72.488 | 1.667 |
| 30 | Early Diapause | Larvae | V11 | 11 | 10 | 23010 | 83 | 85.272 | 1.571 |
| 31 | Early Diapause | Larvae | V11 | 13 | 10 | 18252 | 73 | 88.308 | 2.548 |
| 32 | Early Diapause | Larvae | V12 | 11 | 10 | 19333 | 84 | 76.418 | 2.071 |
| 33 | Early Diapause | Larvae | V12 | 13 | 10 | 16347 | 41 | 40.775 | 0.348 |
| 34 | Early Diapause | Larvae | V14 | 11 | 10 | 20113 | 92 | 86.251 | 2.467 |
| 35 | Early Diapause | Larvae | V14 | 13 | 10 | 31689 | 107 | 85.056 | 1.187 |
| 36 | Early Diapause | Larvae | V15 | 11 | 10 | 16330 | 70 | 63.520 | 1.753 |
| 37 | Early Diapause | Larvae | V15 | 13 | 10 | 29293 | 91 | 78.358 | 1.997 |
| 38 | Early Diapause | Larvae | V16 | 11 | 10 | 25923 | 81 | 62.649 | 0.747 |
| 39 | Early Diapause | Larvae | V17 | 13 | 10 | 27444 | 49 | 45.485 | 0.192 |
| 40 | 1 Month Diapause | Larvae | V02 | 11 | 10 | 26493 | 45 | 33.380 | 1.220 |
| 41 | 1 Month Diapause | Larvae | V04 | 11 | 10 | 16668 | 34 | 40.367 | 2.415 |
| 42 | 1 Month Diapause | Larvae | V07 | 11 | 10 | 23890 | 41 | 45.045 | 0.878 |
| 43 | 1 Month Diapause | Larvae | V08 | 18 | 10 | 17771 | 64 | 70.042 | 2.403 |
| 44 | 1 Month Diapause | Larvae | V09 | 12 | 10 | 28913 | 74 | 64.953 | 2.739 |
| 45 | 1 Month Diapause | Larvae | V10 | 12 | 10 | 20029 | 49 | 44.265 | 1.181 |
| 46 | 1 Month Diapause | Larvae | V11 | 11 | 10 | 19626 | 61 | 51.966 | 1.068 |
| 47 | 1 Month Diapause | Larvae | V12 | 15 | 10 | 24076 | 76 | 61.532 | 0.972 |
| 48 | 1 Month Diapause | Larvae | V14 | 11 | 10 | 29168 | 53 | 33.693 | 1.065 |
| 49 | 1 Month Diapause | Larvae | V15 | 11 | 10 | 24662 | 53 | 54.070 | 2.100 |
| 50 | 1 Month Diapause | Larvae | V16 | 12 | 10 | 27805 | 35 | 27.250 | 2.246 |

**Supplementary Table 3 (cont.)** Overview of the 138 amplicon pools used in this work

| **Pool No** | **Condition** | **Developmental Stage** | **Mother** | **Age of Mother (Days)** | **No of individuals** | **No of Reads^a^** | **No of OTUs^a^** | **Richness and diversity estimates^b^** | |
| --- | --- | --- | --- | --- | --- | --- | --- | --- | --- |
|  |  |  |  |  |  |  |  | **Chao 1** | **Shannon** |
| 51 | 1 Month Diapause | Larvae | V17 | 15 | 10 | 23520 | 55 | 47.797 | 1.361 |
| 52 | 2 Months Diapause | Larvae | V02 | 14 | 10 | 31270 | 63 | 45.270 | 1.178 |
| 53 | 2 Months Diapause | Larvae | V04 | 16 | 10 | 30235 | 67 | 54.488 | 1.338 |
| 54 | 2 Months Diapause | Larvae | V07 | 15 | 10 | 22791 | 36 | 28.858 | 0.234 |
| 55 | 2 Months Diapause | Larvae | V08 | 21 | 10 | 24742 | 19 | 18.033 | 0.217 |
| 56 | 2 Months Diapause | Larvae | V09 | 14 | 10 | 17367 | 53 | 57.767 | 2.434 |
| 57 | 2 Months Diapause | Larvae | V10 | 15 | 10 | 23333 | 43 | 46.833 | 0.613 |
| 58 | 2 Months Diapause | Larvae | V11 | 13 | 10 | 20784 | 56 | 47.490 | 1.497 |
| 59 | 2 Months Diapause | Larvae | V12 | 11 | 10 | 20401 | 55 | 56.934 | 1.972 |
| 60 | 2 Months Diapause | Larvae | V14 | 14 | 10 | 12586 | 46 | 69.900 | 2.185 |
| 61 | 2 Months Diapause | Larvae | V15 | 14 | 10 | 22359 | 37 | 32.175 | 0.307 |
| 62 | 2 Months Diapause | Larvae | V16 | 15 | 10 | 7478 | 55 | 73.374 | 3.513 |
| 63 | 2 Months Diapause | Larvae | V17 | 17 | 10 | 18163 | 43 | 37.660 | 1.540 |
| 64 | 3 Months Diapause | Larvae | V04 | 18 | 12 | 28106 | 42 | 28.858 | 0.542 |
| 65 | 3 Months Diapause | Larvae | V07 | 20 | 12 | 24987 | 76 | 72.570 | 1.979 |
| 66 | 3 Months Diapause | Larvae | V08 | 22 | 12 | 19903 | 82 | 85.271 | 1.854 |
| 67 | 3 Months Diapause | Larvae | V09 | 18 | 12 | 24886 | 47 | 47.008 | 1.453 |
| 68 | 3 Months Diapause | Larvae | V10 | 21 | 12 | 22797 | 62 | 69.830 | 1.341 |
| 69 | 3 Months Diapause | Larvae | V11 | 22 | 12 | 15689 | 41 | 43.080 | 0.851 |
| 70 | 3 Months Diapause | Larvae | V12 | 21 | 12 | 25563 | 50 | 44.638 | 0.865 |
| 71 | 3 Months Diapause | Larvae | V14 | 21 | 12 | 26562 | 76 | 63.054 | 1.699 |
| 72 | 3 Months Diapause | Larvae | V15 | 17 | 12 | 27909 | 76 | 56.109 | 1.047 |
| 73 | 3 Months Diapause | Larvae | V16 | 18 | 12 | 20610 | 61 | 58.233 | 2.947 |
| 74 | 3 Months Diapause | Larvae | V17 | 21 | 12 | 22967 | 54 | 47.635 | 0.482 |
| 75 | 4 Months Diapause | Larvae | V04 | 22 | 12 | 20322 | 57 | 69.438 | 1.585 |

**Supplementary Table 3 (cont.)** Overview of the 138 amplicon pools used in this work

| **Pool No** | **Condition** | **Developmental Stage** | **Mother** | **Age of Mother (Days)** | **No of individuals** | **No of Reads^a^** | **No of OTUs^a^** | **Richness and diversity estimates^b^** | |
| --- | --- | --- | --- | --- | --- | --- | --- | --- | --- |
|  |  |  |  |  |  |  |  | **Chao 1** | **Shannon** |
| 76 | 4 Months Diapause | Larvae | V07 | 24 | 12 | 5589 | 19 | 30.983 | 0.252 |
| 77 | 4 Months Diapause | Larvae | V10 | 24 | 12 | 16353 | 68 | 71.675 | 2.605 |
| 78 | 4 Months Diapause | Larvae | V11 | 26 | 12 | 21993 | 41 | 39.442 | 0.980 |
| 79 | 4 Months Diapause | Larvae | V12 | 24 | 12 | 16231 | 47 | 45.865 | 1.786 |
| 80 | 4 Months Diapause | Larvae | V14 | 25 | 12 | 12772 | 87 | 99.870 | 2.985 |
| 81 | 4 Months Diapause | Larvae | V15 | 21 | 12 | 15445 | 57 | 63.873 | 2.281 |
| 82 | 4 Months Diapause | Larvae | V16 | 21 | 12 | 7662 | 56 | 71.495 | 2.402 |
| 83 | 4 Months Diapause | Larvae | V17 | 24 | 12 | 16624 | 66 | 83.498 | 2.071 |
| 84 | 5 Months Diapause | Larvae | V02 | 23 | 12 | 25862 | 55 | 43.203 | 0.809 |
| 85 | 5 Months Diapause | Larvae | V04 | 24 | 12 | 24001 | 55 | 48.813 | 1.266 |
| 86 | 5 Months Diapause | Larvae | V07 | 26 | 12 | 23913 | 56 | 48.393 | 1.673 |
| 87 | 5 Months Diapause | Larvae | V08 | 34 | 12 | 22578 | 68 | 62.545 | 0.975 |
| 88 | 5 Months Diapause | Larvae | V09 | 24 | 12 | 21914 | 72 | 80.106 | 1.960 |
| 89 | 5 Months Diapause | Larvae | V10 | 25 | 11 | 18356 | 79 | 80.816 | 2.282 |
| 90 | 5 Months Diapause | Larvae | V11 | 31 | 10 | 11617 | 51 | 49.509 | 2.563 |
| 91 | 5 Months Diapause | Larvae | V12 | 29 | 12 | 27567 | 51 | 35.007 | 1.248 |
| 92 | 5 Months Diapause | Larvae | V14 | 34 | 12 | 28431 | 85 | 83.281 | 1.057 |
| 93 | 5 Months Diapause | Larvae | V15 | 25 | 12 | 24751 | 55 | 47.258 | 1.208 |
| 94 | 5 Months Diapause | Larvae | V16 | 23 | 12 | 20349 | 47 | 60.325 | 1.819 |
| 95 | 5 Months Diapause | Larvae | V17 | 27 | 12 | 21914 | 50 | 60.095 | 1.207 |
| 96 | 6 Months Diapause | Larvae | V02 | 27 | 11 | 23950 | 56 | 43.612 | 1.423 |
| 97 | 6 Months Diapause | Larvae | V04 | 27 | 10 | 24921 | 56 | 57.250 | 0.984 |
| 98 | 6 Months Diapause | Larvae | V07 | 28 | 12 | 12628 | 54 | 55.758 | 2.738 |
| 99 | 6 Months Diapause | Larvae | V08 | 32 | 12 | 20030 | 87 | 83.059 | 2.675 |
| 100 | 6 Months Diapause | Larvae | V09 | 25 | 12 | 23372 | 68 | 75.568 | 1.518 |

**Supplementary Table 3 (cont.)** Overview of the 138 amplicon pools used in this work

| **Pool No** | **Condition** | **Developmental Stage** | **Mother** | **Age of Mother (Days)** | **No of individuals** | **No of Reads^a^** | **No of OTUs^a^** | **Richness and diversity estimates^b^** | |
| --- | --- | --- | --- | --- | --- | --- | --- | --- | --- |
|  |  |  |  |  |  |  |  | **Chao 1** | **Shannon** |
| 101 | 6 Months Diapause | Larvae | V10 | 32 | 12 | 15304 | 84 | 77.049 | 2.162 |
| 102 | 6 Months Diapause | Larvae | V11 | 28 | 11 | 20250 | 25 | 22.342 | 0.888 |
| 103 | 6 Months Diapause | Larvae | V12 | 31 | 12 | 16557 | 84 | 80.781 | 1.731 |
| 104 | 6 Months Diapause | Larvae | V14 | 36 | 12 | 24680 | 47 | 47.405 | 0.665 |
| 105 | 6 Months Diapause | Larvae | V15 | 29 | 12 | 14865 | 76 | 86.004 | 2.876 |
| 106 | 6 Months Diapause | Larvae | V16 | 25 | 12 | 7522 | 86 | 116.587 | 1.264 |
| 107 | 6 Months Diapause | Larvae | V17 | 31 | 12 | 9006 | 23 | 24.383 | 0.944 |

| **Pool No** | **Condition** | **Developmental Stage** | **Mother** | **Gender** | **No of individuals** | **No of Reads^a^** | **No of OTUs^a^** | **Richness and diversity estimates^b^** | |
| --- | --- | --- | --- | --- | --- | --- | --- | --- | --- |
|  |  |  |  |  |  |  |  | **Chao 1** | **Shannon** |
| 108 | No Diapause | Adults | V02 | Females | 4 | 9964 | 63 | 75.692 | 3.034 |
| 109 | No Diapause | Adults | V04 | Females | 2 | 11594 | 61 | 74.215 | 2.986 |
| 110 | No Diapause | Adults | V07 | Females | 3 | 18517 | 81 | 66.063 | 2.463 |
| 111 | No Diapause | Adults | V09 | Females | 2 | 12434 | 72 | 79.102 | 2.418 |
| 112 | No Diapause | Adults | V10 | Males | 2 | 22852 | 106 | 86.784 | 1.484 |
| 113 | No Diapause | Adults | V11 | Females | 5 | 11710 | 106 | 84.167 | 3.389 |
| 114 | No Diapause | Adults | V15 | Females | 6 | 17304 | 68 | 51.660 | 0.640 |
| 115 | No Diapause | Adults | V17 | Females | 3 | 20670 | 80 | 62.748 | 2.000 |
| 116 | 3 Months Diapause | Adults | V09 | Females | 2 | 19823 | 96 | 95.475 | 1.579 |
| 117 | 3 Months Diapause | Adults | V10 | Males | 3 | 18201 | 121 | 103.628 | 2.259 |
| 118 | 3 Months Diapause | Adults | V11 | Females | 3 | 18389 | 118 | 102.321 | 2.184 |
| 119 | 3 Months Diapause | Adults | V17 | Females | 4 | 16049 | 100 | 98.885 | 2.114 |

**Supplementary Table 3 (cont.)** Overview of the 138 amplicon pools used in this work

| **Pool No** | **Condition** | **Developmental Stage** | **Mother** | **Gender** | **No of individuals** | **No of Reads^a^** | **No of OTUs^a^** | **Richness and diversity estimates^b^** | |
| --- | --- | --- | --- | --- | --- | --- | --- | --- | --- |
|  |  |  |  |  |  |  |  | **Chao 1** | **Shannon** |
| 120 | 4 Months Diapause | Adults | V02 | Females | 6 | 17840 | 61 | 55.010 | 1.997 |
| 121 | 4 Months Diapause | Adults | V04 | Females | 6 | 16933 | 87 | 83.802 | 3.841 |
| 122 | 4 Months Diapause | Adults | V09 | Females | 6 | 16138 | 91 | 79.811 | 1.027 |
| 123 | 4 Months Diapause | Adults | V10 | Males | 6 | 12672 | 100 | 109.013 | 2.962 |
| 124 | 4 Months Diapause | Adults | V14 | Females | 4 | 7663 | 72 | 82.466 | 3.752 |
| 125 | 4 Months Diapause | Adults | V15 | Females | 4 | 10442 | 88 | 92.147 | 3.834 |
| 126 | 4 Months Diapause | Adults | V17 | Females | 3 | 8977 | 77 | 93.450 | 3.814 |
| 127 | 5 Months Diapause | Adults | V04 | Females | 5 | 13390 | 82 | 91.568 | 0.930 |
| 128 | 5 Months Diapause | Adults | V09 | Females | 4 | 13673 | 87 | 82.542 | 2.563 |
| 129 | 5 Months Diapause | Adults | V10 | Males | 3 | 12770 | 123 | 126.304 | 3.809 |
| 130 | 5 Months Diapause | Adults | V11 | Females | 4 | 4522 | 106 | 147.233 | 4.949 |
| 131 | 5 Months Diapause | Adults | V14 | Females | 3 | 10878 | 63 | 60.917 | 3.243 |
| 132 | 5 Months Diapause | Adults | V15 | Males | 3 | 7783 | 43 | 42.841 | 2.083 |
| 133 | 6 Months Diapause | Adults | V04 | Females | 2 | 5499 | 66 | 86.032 | 3.788 |
| 134 | 6 Months Diapause | Adults | V07 | Females | 5 | 7077 | 93 | 94.275 | 3.501 |
| 135 | 6 Months Diapause | Adults | V09 | Females | 5 | 5748 | 61 | 82.974 | 3.570 |
| 136 | 6 Months Diapause | Adults | V10 | Males | 4 | 7899 | 43 | 51.475 | 3.530 |
| 137 | 6 Months Diapause | Adults | V14 | Females | 4 | 3286 | 43 | 53.120 | 3.869 |
| 138 | 6 Months Diapause | Adults | V17 | Females | 4 | 4058 | 41 | 78.500 | 3.420 |

**^a^**After removal of OTUs containing < 5 reads

**^b^**Based on random subsampling of 5000 sequences for larvae samples and 4000 sequences for adult samples
